# Illustrating, Quantifying, and Correcting for Bias in Post-hoc Analysis of Gene-Based Rare Variant Tests of Association

**DOI:** 10.3389/fgene.2017.00117

**Published:** 2017-09-14

**Authors:** Kelsey E. Grinde, Jaron Arbet, Alden Green, Michael O'Connell, Alessandra Valcarcel, Jason Westra, Nathan Tintle

**Affiliations:** ^1^Department of Biostatistics, University of Washington Seattle, WA, United States; ^2^Department of Biostatistics, University of Minnesota Minneapolis, MN, United States; ^3^Department of Statistics, Carnegie Mellon University Pittsburgh, PA, United States; ^4^Department of Biostatistics and Epidemiology, University of Pennsylvania Philadelphia, PA, United States; ^5^Department of Statistics, Iowa State University Ames, IA, United States; ^6^Department of Mathematics, Statistics, and Computer Science, Dordt College Sioux Center, IA, United States

**Keywords:** next-generation sequencing, SKAT, burden test, case-control, winner's curse

## Abstract

To date, gene-based rare variant testing approaches have focused on aggregating information across sets of variants to maximize statistical power in identifying genes showing significant association with diseases. Beyond identifying genes that are associated with diseases, the identification of causal variant(s) in those genes and estimation of their effect is crucial for planning replication studies and characterizing the genetic architecture of the locus. However, we illustrate that straightforward single-marker association statistics can suffer from substantial bias introduced by conditioning on gene-based test significance, due to the phenomenon often referred to as “winner's curse.” We illustrate the ramifications of this bias on variant effect size estimation and variant prioritization/ranking approaches, outline parameters of genetic architecture that affect this bias, and propose a bootstrap resampling method to correct for this bias. We find that our correction method significantly reduces the bias due to winner's curse (average two-fold decrease in bias, *p* < 2.2 × 10^−6^) and, consequently, substantially improves mean squared error and variant prioritization/ranking. The method is particularly helpful in adjustment for winner's curse effects when the initial gene-based test has low power and for relatively more common, non-causal variants. Adjustment for winner's curse is recommended for all post-hoc estimation and ranking of variants after a gene-based test. Further work is necessary to continue seeking ways to reduce bias and improve inference in post-hoc analysis of gene-based tests under a wide variety of genetic architectures.

## Introduction

In recent years, numerous gene-based rare variant tests of association (hereafter GBTs) have been proposed that seek to aggregate genotype-phenotype association signals across rare variants within a gene to improve the overall evidence of genotype-phenotype association within a gene of interest (Li and Leal, 2008; Madsen and Browning, 2009; Morris and Zeggini, 2010; Price et al., 2010; Zawistowski et al., 2010; Ionita-Laza et al., 2011; Pan and Shen, 2011; Wu et al., 2011; Lee et al., 2012; Greco et al., 2016). These tests can be broadly grouped into two categories: length (burden) tests and joint (variance component) tests based on a geometric interpretation of the hypotheses being tested (Liu et al., 2013). These tests have been motivated by technological advances allowing measurement of rare variants inexpensively and accurately, and by the ongoing search for the genetic basis of many common, complex diseases (Manolio et al., 2009; Schork et al., 2009; Eichler et al., 2010).

For case-control studies considering rare variants, single-marker tests do not yield adequate power. GBTs attempt to address the low power problem by testing the null hypothesis that the population minor allele frequency vector in the cases is the same as the controls for a set of variants within a gene of interest. By simultaneously testing all variants within a gene, multiple testing penalties are reduced as compared to single-marker tests, and power may be improved since aggregating signals from multiple variants associated with the phenotype may lead to improved ability to detect the association. However, a practical problem exists: when GBTs find significant evidence of association with the phenotype, few, if any, standard approaches exist to identify which variant or subset of variants within that gene are truly responsible for increasing or decreasing disease risk, or to provide unbiased estimates of the true effect of these variants.

To date, most work has focused on the problem of fine-mapping regions of interest in an independent sample after initially identifying regions as significant in a genome-wide scan in a discovery sample (Maller et al., 2012; Hormozdiari et al., 2014; Li et al., 2015). Approaches for prioritizing variants in a region of interest for further study include filtering of variants based on prior biological information [e.g., non-synonymous, location (exon vs. intron), predicted deleterious effects (Phen Score)] (Zhan and Liu, 2015), methods designed primarily for common variants (Hormozdiari et al., 2014), and, more recently, Bayesian approaches. Bayesian approaches include those that yield posterior probabilities that single nucleotide polymorphisms (SNPs) are associated with the phenotype (Larson et al., 2016), a risk index for each SNP under the assumption that there is a single causal variant (Maller et al., 2012; Beecham et al., 2013) or allowing for an arbitrary number of causal variants in each region of interest (Quintana et al., 2011). To date, few, if any, rigorously developed frequentist methods exist to identify the most likely causal variant(s) in a gene after a GBT—what we call “post-hoc analysis of a GBT.” In addition to the potentially low power of any single-marker post-hoc rare variant strategy, another compounding issue is that of winner's curse. Winner's curse—also referred to as regression to the mean or selection bias—is bias in parameter estimates after the identification of a statistically significant association (Liu and Leal, 2012). It has been attributed to using the same data for both significance testing and parameter estimation (Xu et al., 2011), and occurs, for example, when parameters are only estimated for the most highly ranked markers (Tan et al., 2014). When testing common genetic variants (e.g., GWAS), research has shown that as power increases, bias in parameter estimates decreases (Xiao and Boehnke, 2009). Recent work (Liu and Leal, 2012) has documented the phenomenon in estimates of average genetic effect after GBTs, but does not explore potential bias in single-marker test statistics, which is of particular concern given the low power of analysis strategies involving rare variants.

Three general classes of methods exist for addressing and correcting winner's curse in single-marker tests of common variants. Replication (independent, split-) sample approaches propose that parameter estimates be obtained from a different sample than was used to test significance. These approaches yield unbiased parameter estimates by looking at an independent replication sample (Goring et al., 2001; Bowden and Dudbridge, 2009), or splitting the initial sample (Sun and Bull, 2005; Faye et al., 2011; Poirier et al., 2015), in each case using one sample for testing and the other for parameter estimation. However, the sample-splitting methods reduce power since not all of the data is being used to assess significance (Sun and Bull, 2005), and the practicalities (time, money) involved in obtaining a replication sample can be prohibitive for many research groups (Liu and Leal, 2012). Furthermore, while approaches involving an independent replication sample may be viable for common variants, they may not be plausible for rare variants. For example, many rare variants are population-specific (Gravel et al., 2011), which leads to the potential of not observing rare variants in either the discovery or the replication samples. Likelihood-based bias correction methods also exist for common variants (Zollner and Pritchard, 2007; Ghosh et al., 2008; Zhong and Prentice, 2008; Xiao and Boehnke, 2009), though recent discussion of these approaches for rare variants have been concerned with challenges in computational tractability of power calculations and the impact of these limitations on resulting correction methods (Liu and Leal, 2012). Finally, there exist bootstrap resampling bias correction methods, which use bootstrap resampling to estimate the bias of a “naïve” (winner's-curse-afflicted) estimator and then define a bias-corrected estimator by subtracting the estimated bias from the naïve estimator. Many variations of this general bootstrap resampling bias correction framework have been proposed and applied in the context of estimation of single-marker effect sizes for common variants (Sun and Bull, 2005; Yu et al., 2007; Sun et al., 2011; Xu et al., 2011; Faye et al., 2013; Zhou and Wright, 2015), and more recently to estimation of the average genetic effect for a GBT (Liu and Leal, 2012). Since the work of Liu and Leal (2012) focuses on estimating and correcting for the overall bias of the entire gene's effect on the phenotype, to date no evaluation of the presence of winner's curse or the application of this bootstrap bias correction framework (or any other winner's curse correction strategy) has been documented in the important context of *post-hoc* estimation of single-marker effect sizes for *rare and common* variants.

In this paper, we document the presence of winner's curse on naïve post-hoc strategies for single-marker tests performed after significant GBTs. Using both analytic methods and simulation we confirm the presence of the bias, quantify it, and establish the key factors related to this bias. We then propose a bootstrap resampling and estimation strategy that adjusts the estimates of individual rare variant effects and leads to improved post-hoc variant prioritization.

## Methods

### Two-step approach

We propose a two-step approach to conducting post-hoc analysis of a GBT (gene-based rare variant test of association).

Step 1: Apply a GBT of genotype–phenotype association to a set of variants of interest. Often the set of variants of interest will be the set of all variants within a gene. Prior work has shown that most GBTs can be classified into one of two broad classes of tests: length (burden; collapsing; linear) or joint (variance components; quadratic) (Liu et al., 2013). We implemented one burden test (*Q*_*burden, weighted*_, or *Q*_*bw*_ for short) and one variance components test (*Q*_*SKAT, weighted*_, or *Q*_*sw*_ for short) in our analysis, selected in order to represent these two broad classes of testing methods. We used the SKAT R package to conduct all tests (Lee et al., 2016) using asymptotic properties of the tests to determine statistical significance. In particular, as implemented in the SKAT R package, $Q_{bw}\approx \;\overset{m}{\underset{i=1\;}{\sum }}w_{i}D_{i}$ and $Q_{sw}\approx \overset{m}{\underset{i=1\;}{\sum }}w_{i}D_{i}^{2}$, where $D_{i}=f_{i}^{+}-f_{i}^{-}$ is the difference between the minor allele frequencies among cases (+) and controls (−) for variant *i*, and there are *m* variants in the gene of interest. Following others (Wu et al., 2011), we used variant weights based on a beta distribution density function with parameters 1 and 25: *w*_*i*_ = $25{\left(1-f_{i}\right)}^{24}$ where *f*_*i*_ is the population minor allele frequency for variant *i*, for both *Q*_*bw*_ and *Q*_*sw*_.

Step 2: Post-hoc analysis. For all genes yielding a significant *p*-value in Step 1, (*p* < α), we considered single-marker, variant level statistics, ${\hat{T}}_{i}$: (1) ${\hat{D}}_{i}={\hat{f}}_{i}^{+}-{\hat{f}}_{i}^{-}$ (the difference in minor allele frequencies) and (2) ${\hat{D}}_{i}^{2}={\left({\hat{f}}_{i}^{+}-{\hat{f}}_{i}^{-}\right)}^{2}$. These variant level statistics, *T*_*i*_, are functions of the observed minor allele frequency in the cases (${\hat{f}}_{i}^{+}=\;{\hat{f}}_{cases,i}=\frac{C_{i}^{+}}{2N^{+}}$) and controls (${\hat{f}}_{i}^{-}={\hat{f}}_{controls,i}=\frac{C_{i}^{-}}{2N^{-}}$), where *N*^+^ and *N*^−^ are the total number of cases and controls in the sample, respectively, and $C_{i}^{+}$ and $C_{i}^{-}$ are the number of minor alleles observed in the cases and controls, respectively.

### General framework for understanding the behavior of post-hoc approaches

Single-marker statistics at Step 2, which are a function of observed single-marker minor allele frequencies, often perform less than optimally (e.g., they are biased; see the *Results* Section). In the *Appendix* (in the Supplementary Material; Sections A.1 and A.1.1 for a general argument, and A.2 for an argument specifically relevant to our simulation study) we provide a detailed demonstration that, under a set of weak assumptions, a Step 1 GBT (*Q*) followed by a single-marker statistic *T*_*i*_ leads to the following relationship for $Bias\left({\hat{T}}_{i}\right)$, the observed bias of the post-hoc statistic ${\hat{T}}_{i}$ at variant *i*:

(1) $$\text{Bias}\left({\hat{T}}_{\text{i}}\right)=\;\frac{\text{Bias}\left(Q\right)}{V\left(Q\right)}\left(w_{i}\text{Var}\left(T_{i}\right)+\sum_{j\neq i}w_{j}\text{Cov}(T_{i},T_{j})\right).$$

This relationship demonstrates that the bias of the naïve Step 2 statistic can be decomposed into an overall gene bias/variance term, and then a unique term for each variant in the gene. The variant-specific term depends on the minor allele frequency and relative risk of the variant, the choice of weights for the GBT, and the strength of LD between the variant of interest and other variants in the gene. The overall gene bias term in Equation (1) is the *observed* bias of the GBT (i.e., for a given, observed value of the test statistic *Q*), and due to winner's curse will often be quite large when we perform post-hoc estimation only after a significant Step 1 test. This in turn will result in a large post-hoc bias in single-marker statistics ${\hat{T}}_{i}$.

### Bootstrap resampling bias correction approach

Recently, a bootstrap resampling approach was proposed to estimate and adjust for bias in single-marker tests on common variants in GWAS studies (Sun and Bull, 2005). An extension of this approach for GBTs calculates an unbiased estimate of the average genetic effect at the locus of interest (Liu and Leal, 2012). Our proposed approach is in the spirit of these earlier efforts, but applied to GBTs, with a goal of attaining unbiased post-hoc estimates for single variants.

The three-part bias correction approach is as follows:

Part 1. Calculate the GBT of interest, *Q*, on the sample of *N* = *N*^+^ + *N*^−^ subjects across a set of *m* variants of interest (often, all variants in a gene). If the test yields a significant association statistic (*p*_*Q*_ < α) continue to Step 2. Otherwise, stop: there is no need for post-hoc single-marker analysis.

Part 2. Calculate naïve single-marker association statistics ${\hat{T}}_{i}$ for all variants, *i* = 1, …, *m*. These statistics will frequently be biased toward showing stronger association than is actually present in the population (due to winner's curse).

Part 3. Take *b* = 1, …, *B* bootstrap resamples of the *N* subjects, separately resampling cases and controls so that each bootstrap sample again has *N*^+^ cases and *N*^−^ controls. This will yield $N_{b}^{*}$ subjects occurring at least once in the *b* ^*th*^ bootstrap sample and $N_{b}^{R}=N-N_{b}^{*}$ not present in the *b* ^*th*^ bootstrap sample (the residual sample). Note that, on average, $N_{b}^{R}\approx \left(\frac{1}{3}N\right)$. For each bootstrap sample, compute the GBT, $Q_{b}^{*}$, using the same gene-based rare variant test *Q* as in Part 1. If the GBT is significant on the bootstrap sample, compute the same single-marker post-hoc statistics used in Part 2 on the bootstrap (${\hat{T}}_{i_{b}}^{*}$) and residual (${\hat{T}}_{i_{b}}^{R}$) samples. The bias in the single-marker test statistic ${\hat{T}}_{i}$ can then be estimated for each bootstrap sample as ${\hat{Bias}}_{b}\left({\hat{T}}_{i}\right)={\hat{T}}_{i_{b}}^{*}-{\hat{T}}_{i_{b}}^{R},b=1,\ldots ,B$.

In general, the distribution of ${\hat{Bias}}_{b}\left({\hat{T}}_{i}\right)$ across the bootstrap samples *b* = 1, …, *B* can be used to adjust for the bias of the naïve statistics ${\hat{T}}_{i}$: ${\hat{T}}_{i,adjusted}={\hat{T}}_{i}-f\left({\hat{Bias}}_{b}\left({\hat{T}}_{i}\right)\right)$, where *f* could be the mean, median or other univariate summary statistic summarized across the significant ($p_{Q_{b}^{*}}<\alpha$) bootstrap samples. In our analyses we followed the spirit of Liu and Leal (2012) and calculated our bias-adjusted estimates using

$${\hat{T}}_{i,adjusted}=\left\{ \begin{array}{l} \max\left({\hat{T}}_{i}-\underset{b\;:\;p_{Q_{b}^{\text{*}}}<\alpha }{\text{median}}\left({\hat{Bias}}_{b}({\hat{T}}_{i})\right),\;0\right)\text{ if }{\hat{T}}_{i}>0\\ \min\left({\hat{T}}_{i}-\underset{b\;:\;p_{Q_{b}^{\text{*}}}<\alpha }{\text{median}}\left({\hat{Bias}}_{b}({\hat{T}}_{i})\right),\;0\right)\text{ if }{\hat{T}}_{i}<\;0. \end{array}\right.$$

**Figure 1:**
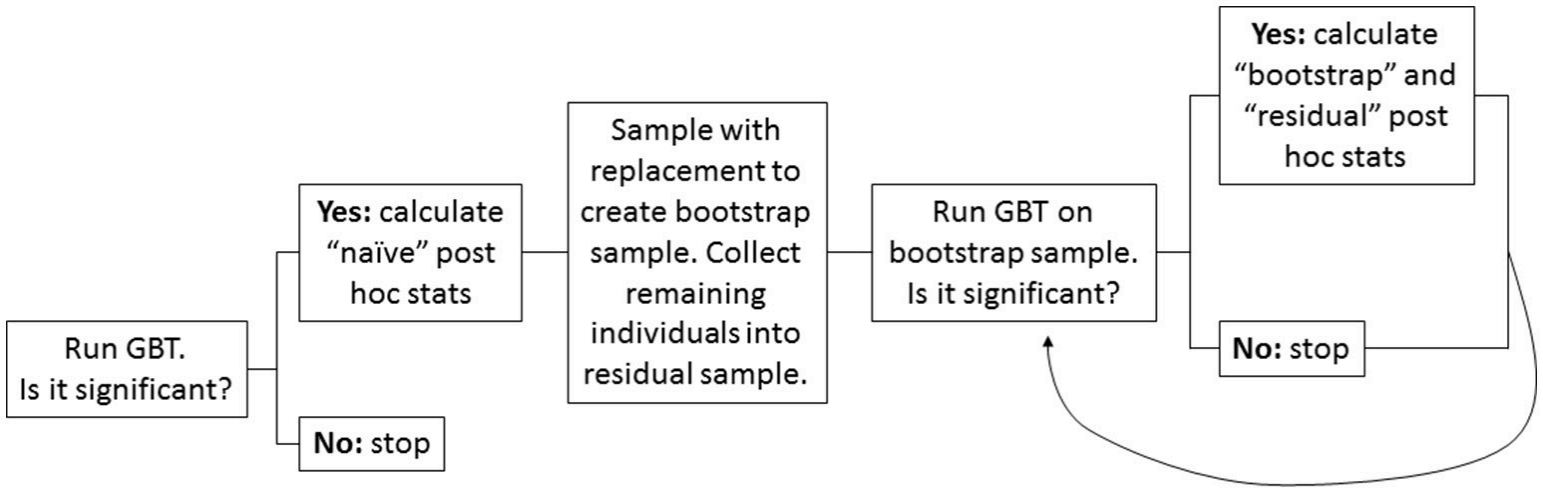
Flow chart depicting the bootstrap resampling approach. A high-level overview of the steps involved in the proposed bootstrap resampling approach to adjust for winner's curse in post-hoc single variant analyses.

Figure 1 provides a visual overview of the bootstrap resampling approach.

### Simulated data

Our theoretical work in the *General Framework for Understanding the Behavior of Post-hoc Approaches* Section demonstrates the behavior of post-hoc single-marker test statistics under very general assumptions. However, in order to illustrate this behavior and to evaluate the performance of our proposed bootstrap resampling bias correction approach we conducted a simulation study under a simpler set of scenarios. Our simulation study considered 50 different combinations of minor allele frequencies and relative risks. We also considered 10 different distributions of minor allele frequencies *f* across sets of 10 variants (representing one “gene”): (a) All 10 SNPs are “rare” (two levels resulting from either all *f* = 0.01% or all *f* = 0.1%), (b) one common neutral variant and nine rare variants (four levels resulting from all combinations of common variant *f* = 5% or *f* = 1% and rare variants *f* = 0.01% or *f* = 0.1%) or (c) two common (one neutral; one risk increasing) variants and eight rare variants (four levels resulting from all combinations of common variant *f* = 5% or *f* = 1% and rare variants *f* = 0.01% or *f* = 0.1%). For each distribution of minor allele frequencies we considered five different relative risk distributions (% variants Risk Increasing: % Neutral: % Risk Decreasing): (1) 20:80:0, (2) 50:50:0, (3) 80:20:0, (4) 20:60:20, and (5) 0:100:0, for a total of 50 different combinations. Relative risk λ for all risk variants is determined by the minor allele frequency, *f*. Namely, for risk increasing variants we used the following combinations of (*f*, λ)*:* (5%, 1.2), (1%, 1.5), (0.1%, 2) and (0.01%, 8). For risk reducing variants we used (5%, 1/1.2), (1%, 1/1.5), (0.1%, 1/2) and (0.01%, 1/8). For all simulations we used 1,500 cases and 1,500 controls, and simulated all variants independently. We performed 10,000 simulations for each of the 50 simulation settings (see Supplementary Table 1 for details on each of the 50 simulation settings).

### Analysis of simulated data

We applied Step 1 and Step 2 (see the *Two-Step Approach* Section) to each simulated data set. In particular, we computed *p*-values for each GBT *Q*_*bw*_ and *Q*_*sw*_. Step 1 power was computed as the proportion of the 10,000 simulated data sets yielding *p*-values less than the significance level. We considered two different significance levels (α): 0.01 and 0.0001. If the Step 1 test was significant we performed bootstrap resampling (Step 2) for that test: We computed adjusted and unadjusted versions of the post-hoc statistics (${\hat{D}}_{i}$ or ${\hat{D}}_{i}^{2})$ for each simulation that was significant at Step 1. Bias is computed as the average of the difference between the single-marker statistic and its expected value. Expected values for each simulation setting are reported in Supplementary Table 1, and their general form is derived in the *Appendix* (Section A.2). MSE is computed as the average of the squared difference between the estimated single-marker statistic and its expected value.

### Software

Corresponding R functions that implement the bootstrap correction approach proposed here are available at: http://www.dordt.edu/statgen.

## Results

### Changes in bias and MSE

Across 50 simulation settings and four different Step 1% testing situations (both of *Q*_*bw*_ and *Q*_*sw*_ at 1% and 0.01% significance levels; a total of 200 situations), 161 of these 200 situations yielded more than one simulation out of 10,000 with a significant Step 1 result. Averaging across each of the 10 variants in each of these 161 situations showed improvements in the overall bias and MSE of ${\hat{D}}_{i}$ [average bias before adjustment = 5.7 × 10^−4^ vs. average bias after adjustment = 3.1 × 10^−4^, with paired t-test *p* < 2.0 × 10^−16^; MSE: 9.7 × 10^−6^ (unadjusted) vs. 5.5 × 10^−6^ (adjusted) with paired t-test *p* < 2.0 × 10^−16^] and ${\hat{D}}_{i}^{2}$ [average bias: 1.0 × 10^−5^ (unadjusted) vs. 3.7 × 10^−6^ (adjusted) with paired t-test *p* < 2.0 × 10^−16^; MSE: 2.7 × 10^−9^ (unadjusted) vs. 1.1 × 10^−9^ (adjusted) with paired t-test *p* = 2.2 × 10^−16^]. In short, the bias and MSE are, on average, halved across all settings. Thus, on average we have reduced, but not eliminated, the bias in these statistics [both 3.1 × 10^−4^ (average adjusted bias in ${\hat{D}}_{i})$ and 3.7 × 10^−6^ (average adjusted bias in ${\hat{D}}_{i}^{2})$ are significantly different than zero].

Table 1 provides an overall view of improvements to bias and MSE (when at least one variant is causal) for two possible combinations of Step 1 and Step 2 test statistics: *Q*_*bw*_ followed by *D*_*i*_ and *Q*_*sw*_ followed by $D_{i}^{2}$. On average, 84% of variants show reduction in bias and MSE after applying our bootstrap bias correction strategy. Importantly, across all scenarios considered there are considerably more variants helped by our correction strategy than hurt, and the typical magnitude of improvement is substantially larger (2–150 times) than the typical magnitude of decline. In other words, the method substantially reduces the bias (and MSE) for most variants, and for those variants it is not helping, the increase in bias (or MSE) is relatively small. Supplementary Table 2 provides results for all four possible combinations of Step 1 and Step 2 test statistics, and Supplementary Figure 1 provides a complementary visual overview. Results across the 10 simulation settings with no causal variants are contained in Supplementary Table 3 and show a generally similar pattern as in Table 1. In essence, the improvements in bias for null simulation settings can be thought of as making the resulting distribution of post-hoc variant effect estimates “more noisy,” which is good for genes containing no causal variants.

**Table 1:** Overall improvement in bias and MSE of bias-adjusted statistics across 10,000 replications of the 40 alternative hypothesis simulation settings.

**Step 1: GBT**	**Step 2: Single-marker statistic**	**Step 1 sig. level (%)**	**Bias or MSE^a^**	**% of improved variants^b^**	**Median improvement^c^**	**Median decline^d^**	**Median increase/Median decrease^e^**
*Q*_*bw*_	${\hat{D}}_{i}$	1	Bias	0.94	1.63 × 10^−04^	3.14 × 10^−05^	5.20
			MSE	0.90	7.32 × 10^−08^	1.34 × 10^−08^	5.47
		0.01	Bias	0.97	2.10 × 10^−04^	9.48 × 10^−05^	2.22
			MSE	0.97	2.04 × 10^−07^	6.04 × 10^−08^	3.37
*Q*_*sw*_	${\hat{D}}_{i}^{2}$	1	Bias	0.64	2.37 × 10^−07^	2.06 × 10^−08^	11.51
			MSE	0.77	1.11 × 10^−12^	1.68 × 10^−14^	65.95
		0.01	Bias	0.69	4.85 × 10^−07^	2.81 × 10^−08^	17.29
			MSE	0.81	2.85 × 10^−12^	1.92 × 10^−14^	148.22

*Results shown for two pairs of Step 1 and Step 2 test statistics: Q_bw_ followed by D_i_ and Q_sw_ followed by $D_{i}^{\text{2}}$*.

a *Bias is computed as the average difference between the estimated single-marker post-hoc statistic and its expected value. MSE is computed as the average squared difference between the estimated single-marker post-hoc statistic and its expected value*.

b *Computed as the percent of variants for which bias (or MSE, depending on the row of the table) decreased after implementing our bootstrap bias-correction strategy*.

c *The median change in bias (or MSE) among variants that show an improvement after adjustment (i.e., bias (or MSE) is smaller after adjustment)*.

d *The median change in bias (or MSE) among variants that show a decline after adjustment (i.e., bias (or MSE) is larger after adjustment)*.

e *The ratio of the previous two columns*.

**Figure 2:**
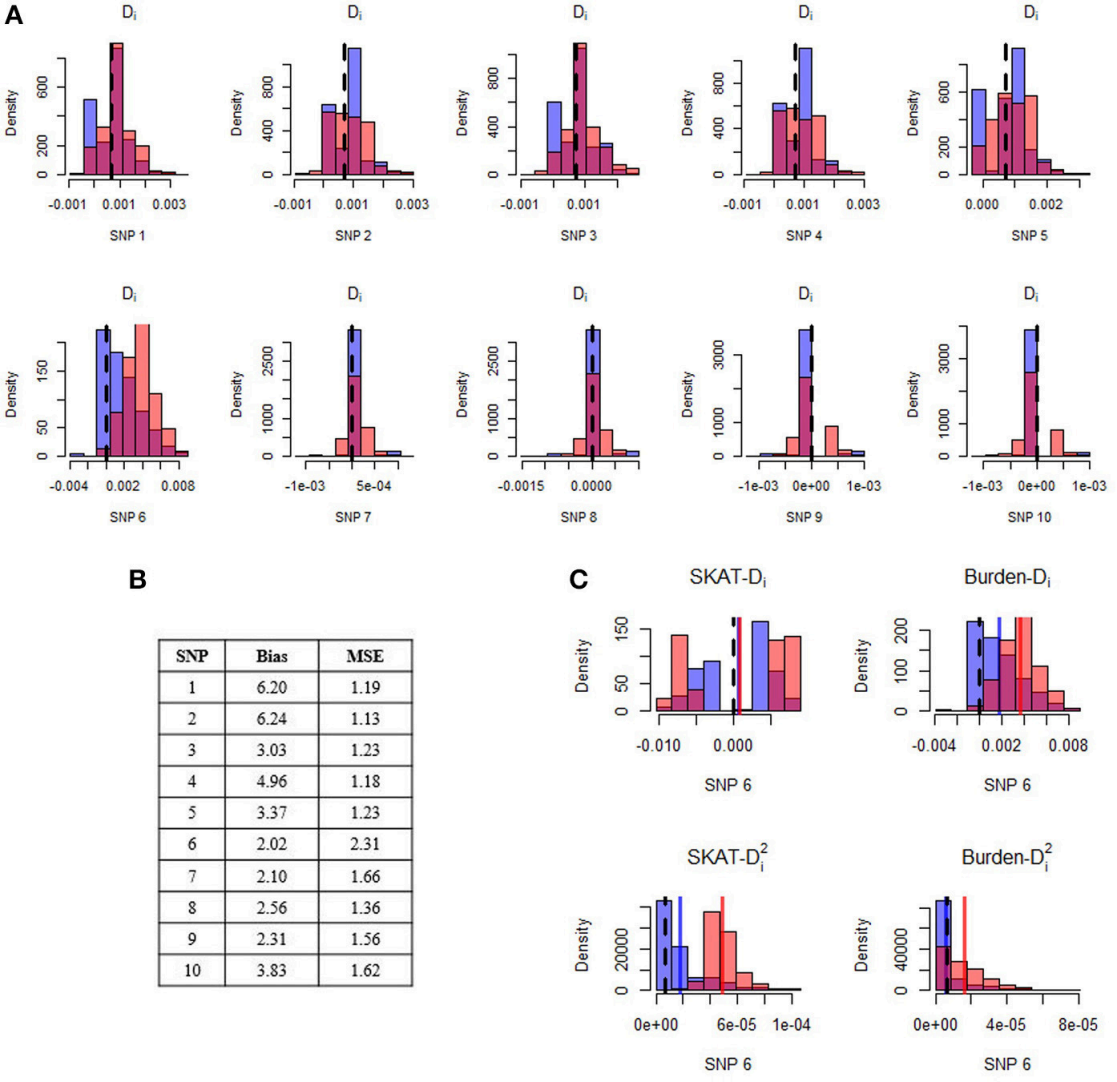
Distribution of estimated difference in minor allele frequencies before and after application of the post-hoc adjustment strategy. Panel **(A)** displays histograms of naive (light red) and bias-corrected (blue) differences in minor allele frequencies for each SNP in a single simulated gene. The dark pink color is the overlap of the two histograms. The black dashed line represents the expected difference in minor allele frequencies. Results are shown for post-hoc analyses conducted after conducting a burden test with α = 0.01 at Step 1. The top row of SNPs (1–5) are risk-increasing with relative risk of 8, and the bottom row of SNPs (6–10) are neutral. All SNPs are very rare (MAF = 0.0001), with the exception of SNP 6 which has minor allele frequency of 0.01. The Step 1 power of this gene was 11.7% across 10,000 replications. Panel **(B)** displays the relative improvement in bias and mean squared error of the bias-corrected post-hoc statistic for each SNP, where relative improvement is computed as the estimated bias (MSE) before adjustment divided by the estimated bias (MSE) after adjustment. Panel **(C)** illustrates the results from SNP6. In particular, we show results for the post-hoc difference in minor allele frequencies after conducting *Q*_*sw*_ (top left) and *Q*_*bw*_ (top right) tests with α = 0.01 at Step 1, as well as results for the post-hoc squared difference in minor allele frequencies after conducting *Q*_*sw*_ (bottom left) and *Q*_*bw*_ (bottom right) test with α = 0.01 at Step 1.

Figure 2 illustrates a specific simulation setting with five rare (MAF = 0.01%) risk-increasing (relative risk = 8) SNPs, four rare (MAF = 0.01%) neutral (relative risk = 1) SNPs, and one more common (MAF = 1%) neutral SNP. In this setting, all variants show improvement, with relative improvement in bias between two- and six-fold and relative improvement in MSE between 1.1- and 2.3-fold after implementation of our bias correction strategy. In particular, we note that the common neutral variant (“SNP 6”) has the largest reduction in MSE, representing a decrease in both bias and variance. Across all combinations of Step 1 tests and Step 2 post-hoc statistics (depicted for SNP 6 in Figure 2C), our bias-corrected statistics are closer on average to their expected value than the unadjusted/naïve statistics (i.e., they are less biased). The improvement of our bias-correction approach is especially evident for SNP 6 when we follow up *Q*_*bw*_ with ${\hat{D}}_{i}$ or *Q*_*sw*_ with ${\hat{D}}_{i}^{2}$. However, across all SNPs we see that even when bias is not reduced by much, our bias correction strategy still reduces MSE, reflecting a small reduction in bias accompanied by an additional reduction in variance.

**Figure 3:**
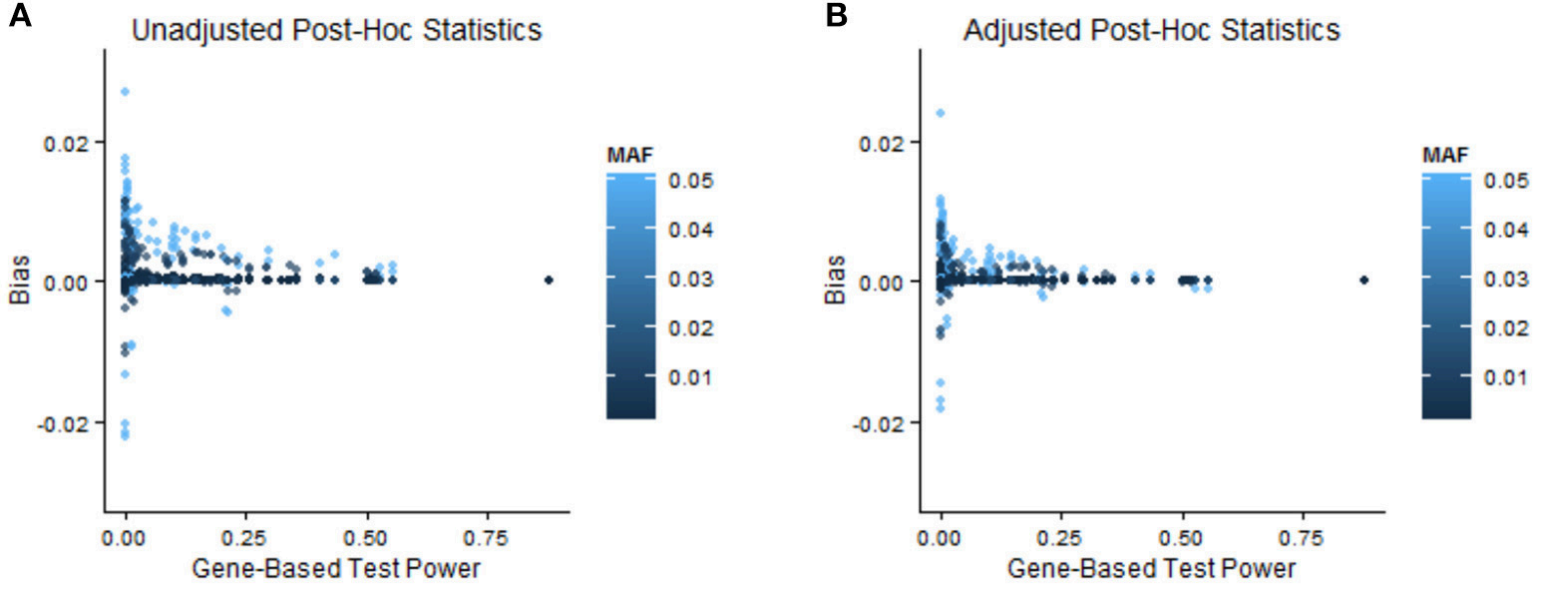
Relationship between bias, minor allele frequency, and GBT power. These scatterplots show the bias of unadjusted **(A)** and adjusted **(B)** single-marker post-hoc statistics vs. the power of the GBT in which that variant is contained. All combinations of Step 1 test, Step 2 single-marker statistic, and significance level are shown for all SNPs and all simulation settings, so a total of 4,000 (=2^*^2^*^2^*^10^*^50) points are in each scatterplot. The points are colored by the MAF of the variant, with lighter colors corresponding to larger MAF.

In order to illustrate when bias adjustments are more or less beneficial, we stratify the results by power of the GBT and by MAF and relative risk of the variant. In the *Appendix* (especially A.1.1 and A.1.3) we discuss the impact of GBT power on bias and MSE of naïve post-hoc single-marker test statistics. Specifically, when GBT power is low we expect to see more bias, and as a result it seems that with low GBT power there is correspondingly more room for improvement in bias. In Table 2 (two pairs of Step 1 and Step 2 statistics) and Supplementary Table 4 (all pairs of Step 1 and Step 2 statistics), we see that the magnitude of improvement in bias or MSE was often larger when GBT power was low (<0.2) compared to when it was high (≥0.2). Interestingly, though, this is not always true, and in many instances our bootstrap bias correction provides improvement for a similar proportion of variants across levels of GBT power. Supplementary Table 5 stratifies the simulation results by both minor allele frequency and relative risk. In general, relatively more common variants with larger relative risks were helped by the bias correction procedure (more were adjusted in the correct direction and the relative amount of adjustment was better). Figure 3 provides a corresponding visual illustration. In both panels we see that the scatterplots have a funnel shape, which shows us that the bias of post-hoc single-marker statistics (unadjusted or adjusted) tends to decrease with increasing power of the Step 1 test. We also see that the variants with larger bias tend to have larger MAF. Furthermore, in comparing the two panels (unadjusted vs. adjusted) we see that the bias of the adjusted post-hoc statistics tends to be smaller than the bias of the unadjusted post-hoc statistics, as all points in the right panel are shrunk toward zero.

**Table 2:** Improvement in bias and MSE of bias-adjusted statistics stratified by the power of the Step 1 test.

**Step 1: GBT**	**Step 2: Single-marker statistic**	**Step 1 sig. level (%)**	**Bias or MSE^a^**	**Step 1 power**	**% of improved variants^b^**	**Median improvement^c^**	**Median decline^d^**	**Median increase/Median decrease^e^**
*Q*_*bw*_	${\hat{D}}_{i}$	1	Bias	0−0.05	0.90	1.77 × 10^−04^	3.24 × 10^−05^	5.45
				0.05−0.2	1.00	1.76 × 10^−04^	–	–
				0.2−0.5	1.00	1.52 × 10^−04^	–	–
				0.5−1	0.85	1.51 × 10^−04^	1.67 × 10^−05^	9.04
			MSE	0−0.05	0.99	1.89 × 10^−07^	1.07 × 10^−09^	177
				0.05−0.2	0.96	6.97 × 10^−08^	1.13 × 10^−08^	6.16
				0.2−0.5	0.90	3.79 × 10^−08^	1.28 × 10^−08^	2.96
				0.5−1	0.60	4.66 × 10^−08^	3.52 × 10^−08^	1.32
		0.01	Bias	0−0.05	0.96	2.09 × 10^−04^	9.48 × 10^−05^	2.21
				0.05−0.2	1.00	2.67 × 10^−04^	–	–
				0.2−0.5	1.00	1.83 × 10^−04^	–	–
				0.5−1	–	–	–	–
			MSE	0−0.05	0.96	2.25 × 10^−07^	6.04 × 10^−08^	3.72
				0.05−0.2	1.00	2.15 × 10^−07^	–	–
				0.2−0.5	1.00	5.10 × 10^−08^	–	–
				0.5−1	–	–	–	–
*Q*_*sw*_	${\hat{D_{i}}}^{2}$	1	Bias	0−0.05	0.68	1.49 × 10^−07^	1.73 × 10^−08^	8.60
				0.05−0.2	0.62	4.75 × 10^−07^	2.15 × 10^−08^	22.1
				0.2−0.5	0.55	2.43 × 10^−07^	2.06 × 10^−08^	11.8
				0.5−1	–	–	–	–
			MSE	0−0.05	0.74	9.76 × 10^−13^	1.37 × 10^−14^	71.4
				0.05−0.2	0.81	2.45 × 10^−12^	1.87 × 10^−14^	131
				0.2−0.5	0.77	1.52 × 10^−12^	1.85 × 10^−14^	82.4
				0.5−1	–	–	–	–
		0.01	Bias	0−0.05	0.69	4.85 × 10^−07^	2.81 × 10^−08^	17.3
				0.05−0.2	–	–	–	–
				0.2−0.5	–	–	–	–
				0.5−1	–	–	–	–
			MSE	0−0.05	0.81	2.85 × 10^−12^	1.92 × 10^−14^	148
				0.05−0.2	–	–	–	–
				0.2−0.5	–	–	–	–
				0.5−1	–	–	–	–

*Results shown for two pairs of Step 1 and Step 2 test statistics: Q_bw_ followed by D_i_ and Q_sw_ followed by $D_{i}^{\text{2}}$*.

a *Bias is computed as the average difference between the estimated single-marker post-hoc statistic and its expected value. MSE is computed as the average squared difference between the estimated single-marker post-hoc statistic and its expected value*.

b *Computed as the percent of variants for which bias (or MSE, depending on the row of the table) decreased after implementing our bootstrap bias-correction strategy. Set to – if no step 1 tests had power in that range*.

c *The median change in bias (or MSE) among variants that show an improvement after adjustment (i.e., bias (or MSE) is smaller after adjustment). Set to – if no tests in that range*.

d *The median change in bias (or MSE) among variants that show a decline after adjustment (i.e., bias (or MSE) is larger after adjustment). Set to – if no tests in that range or no variants showed decline (i.e., % of improved variants is 1)*.

e *The ratio of the previous two columns. Set to – if either of previous two columns is –*.

### Changes in rank

**Figure 4:**
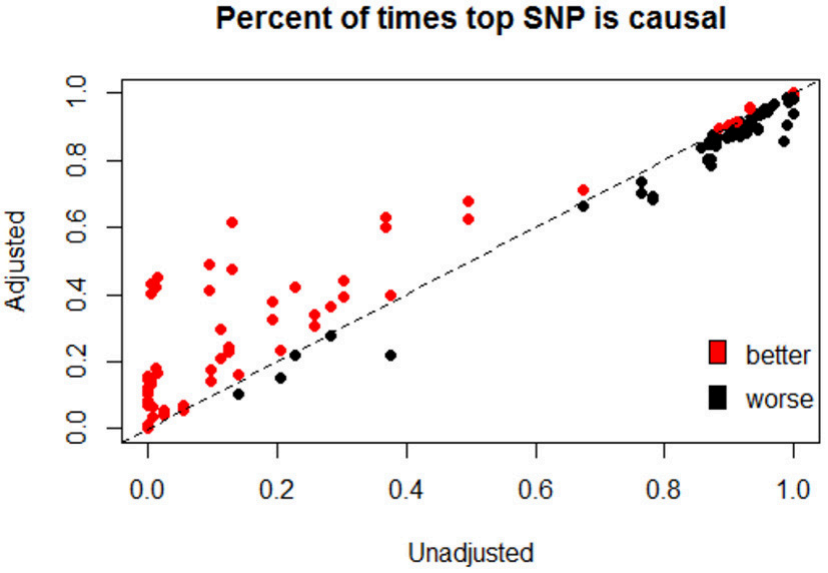
Percent of times that the top ranked SNP based is causal before and after adjustment. After conducting a GBT (Step 1) which yields a significant result, all SNPs in the gene are then ranked by either ${\hat{D}}_{i}$ or ${\hat{D}}_{i}^{2}$, both before and after adjustment. The figure shows the percent of times the top-ranked SNP is causal when ranking is based on the adjusted statistic vs. the unadjusted statistic. Points are colored by whether or not the adjusted statistic provides “better” ranking results, where a “better” ranking result is one in which the top-ranked SNP is causal a higher proportion of the time (across the 10,000 simulation settings). The dashed line is y = x, so that points falling above the line are settings where the adjusted statistics are better (top ranking SNP is more likely to be causal after adjustment), and points falling below the line are settings where the adjusted statistics perform worse (top ranked SNP is less likely to be causal after adjustment). This figure depicts all 40 non-null simulations and all four combinations of Step 1 (*Q*_*sw*_ or *Q*_*bw*_) and Step 2 (${\hat{D}}_{i}$ or ${\hat{D}}_{i}^{2}$) statistics.

We have focused primarily on improvements to the bias and MSE of post-hoc single-marker statistics after our proposed post-hoc adjustment strategy. However, we note that a reasonable approach taken to identify causal variants after a significant GBT involves ranking of individual markers within the gene. Figure 4 illustrates the overall results (as measured by the percent of times the top-ranked variant is causal) of ranking all variants before and after adjustment. We see that ranking improves in many cases, and often substantially, after our bootstrap bias correction approach (as shown by the number of points above the *y* = *x* line, and their distance from that line). The times that ranking based on the adjusted statistics is not better tend to be settings when the percent of times the top SNP is causal was already quite high before adjustment, and the ranking after adjustment is often not that much worse in these cases. Supplementary Figure 2 looks at the percent of times the top two ranked variants are causal, and shows a similar pattern.

Revisiting the simulation setting illustrated in Figure 2 finds that the percent of times that the top ranked SNP is actually causal moves from 13.1% to 47.6% after adjustment. Similarly, the percent of times that the top two SNPs are causal moves from 8.3% before adjustment to 47.6% after adjustment. This dramatic improvement is mainly due to the substantial change in the rank of the more common, non-causal SNP (SNP 6) from an average rank of 1.35 before adjustment to 3.35 after, along with modest improvements to the remaining causal (ranks closer to 1) and non-causal (ranks closer to 10) variants.

## Discussion

We have documented widespread bias in post-hoc single-marker statistics performed after a significant GBT. This bias is due to winner's curse: namely, that the naïve post-hoc single-marker association statistics (e.g., ${\hat{D}}_{i}$ and ${\hat{D}}_{i}^{2}$) overestimate the strength of the true association. Winner's curse is particularly problematic for Step 2 of gene based testing situations when the Step 1 power is low. As demonstrated both theoretically (see *Appendix*, especially A.1.1 and A.1.3, and Supplementary Figures 3, 4) and via simulation, there is a direct relationship between minor allele frequency and bias/MSE, leading unadjusted post-hoc statistics (${\hat{D}}_{i}$ and ${\hat{D}}_{i}^{2})$ to be particularly prone to over-estimation for more common, non-causal variants. Thus, it is possible to yield post-hoc association statistics which not only yield biased effect size estimates, but the differential bias by minor allele frequency can also lead to ranking of non-causal variants ahead of causal variants.

Importantly, any adjustment strategy for these winner's curse effects must account for the fact that a Step 1 test has already been conducted on the same data set. Failing to account for this fact when computing unadjusted variant-association statistics leads to an inflated sense of confidence in the observed associations—exactly the winner's curse problem. Thus, any post-hoc strategy which attempts to identify causal variants must account for the fact that the Step 1 test has been conducted and adjust for this in Step 2. We have proposed a method which attempts to provide unbiased post-hoc estimates of single variant association statistics using a bootstrap approach. By using a bootstrapping approach, estimates of the sample bias can be obtained and used to create unbiased single-marker association statistics. In general, the proposed method improves post-hoc association statistics by making the estimates less biased and yielding better post-hoc mean squared error and ranks. These improved ranks and less-biased estimates of a variant's association with disease are useful to quantify association, plan replication studies and, ultimately, lead to the identification of truly causal variants.

As shown above, the adjustment method tends to perform best when Step 1 power is lowest, exactly when the effects due to winner's curse are largest. In other cases, the adjustment method has little impact (positive or negative) on the post-hoc inference. Furthermore, the choice of post-hoc statistic (Step 2) is important to align with the GBT statistic (Step 1). As others have noted (Liu et al., 2013), different GBTs are testing different null hypotheses and, as such, are looking for particular types of genetic architecture. Thus, we argue that the best approach to the choice of post-hoc, single-marker statistics is to choose a statistic which mirrors the hypothesis being tested at Step 1. If you find evidence of a particular genetic architecture at Step 1, it does not make sense to then go and look for a different type of architecture at Step 2. In particular, if a variance components test is used at Step 1, using a single-marker statistic in the spirit of variance components (${\hat{D}}_{i}^{2}$) at Step 2 is the most natural and consistent choice, whereas a statistic in the spirit of ${\hat{D}}_{i}$ is most appropriate after a burden test. Finally, it is worth noting that the bias of the ${\hat{D}}_{i}^{2}$ statistic is often minimal, with the primary impact of winner's curse occurring on the variance of the statistic (primarily by making the distribution more bimodal—driving it away from zero).

We have focused on the general classes of burden and variance components tests and two relatively simple and straightforward choices of post-hoc statistics (${\hat{D}}_{\text{i}}\;\text{and}\;{\hat{D}}_{i}^{2}$), as has been suggested by others (Xiao and Boehnke, 2009), but it is important to note that our proposed method is quite general. In particular, the general bootstrap bias correction framework we propose is valid for numerous other choices of Step 1 and Step 2 statistics, though some care should be taken to align the two statistics as noted in the previous paragraph. Furthermore, although our simulation study focuses only on these few choices of Step 1 and Step 2 statistics, as well as a case-control phenotype with no LD, our theoretical results apply to a much wider range of settings, including quantitative traits, correlated genotypes, and more (see Section A.1 in the *Appendix* for more details).

There are a variety of areas of potential future work. First, while our preliminary analysis of the method's performance on genotypes in the presence of linkage disequilibrium is promising and shows similar patterns as were observed on independent genotypes (see Supplementary Figure 5; Bickeböller et al., 2014), further work is needed to continue to explore and understand the implications of correlated genotypes on the observed results. In particular, since the proposed bootstrapping strategy is not a model-based approach that capitalizes on LD structure, this may suggest a way to further improve bias correction. However, further work is needed to explore this hypothesis and evaluate performance in data with correlated genotypes. Although further exploration would be useful, it is important to note, however, that our method does not rely on any assumptions of independent markers: our theoretical work suggests that post-hoc bias will still exist in the presence of LD (and may even be exaggerated), and existing literature suggests that this bootstrap resampling bias correction approach should work in the presence of LD (Tan et al., 2014).

Our focus in this paper has been on bias in post-hoc estimation. Initial analyses indicate that post-hoc bias can also lead to problems in downstream analyses, such as variant prioritization. However, further work is needed to assess the impact of post-hoc bias on other possible downstream analyses, such as heritability estimation or calculation of sample sizes for replication studies, and to develop appropriate methods for post-hoc testing in this fundamentally difficult post-selection inference setting. In addition, post-hoc ranking strategies are challenged by extremely rare variants (e.g., singleton; doubletons). Further work is needed to explore these extreme cases since all post-hoc, variant-level association statistics will struggle to yield robust estimates in these cases. We anticipate that there may be ways to combine extremely rare variants in an ad hoc manner to yield post-hoc association statistics for sets of variants which are more robust and reliable in these situations. Further work is necessary here as well.

A number of extensions of the method are possible and worth further exploration including alternative variant weights when conducting post-hoc ranks (either based on MAF or prior biological information), conditioning on known causal variants and approaches which attempt to predict the most likely subset of causal variants vs. a ranked list based on single-marker statistics. An extensive effort will be needed to fully address all of the many issues presented by this setting of post-hoc inference.

Winner's curse is a widespread and well-understood phenomenon for common variants in genetic association studies. However, the use of GBTs for sequence data in order to incorporate rare variants into resulting association tests still leads to substantial winner's curse effects, especially when attempting to prioritize and identify causal variants using single-marker methods (Step 2) after a significant GBT (Step 1). We have shown that low powered Step 1 tests, and relatively more common, non-causal variants are particularly prone to winner's curse effects and can yield substantial bias in resulting estimates of genotype–phenotype association, which can in turn lead to cases where the top ranked variant is non-causal and causal variants show less evidence of association. We have proposed a preliminary method of adjusting for winner's curse when generating post-hoc ranks of variants. Our approach reduces bias and leads to improved variant prioritization. Further work is needed to continue to improve the performance of the proposed method, and to develop further methods which address the plethora of issues presented by this setting of post-hoc inference.

## Author contributions

All authors participated in the design of the study, methods development, and results summarization. KG and NT drafted the initial manuscript. JW led the implementation of the simulations. All authors read and approved the final manuscript.

### Conflict of interest statement

The authors declare that the research was conducted in the absence of any commercial or financial relationships that could be construed as a potential conflict of interest.
