# Supplementary material for: Illustrating, Quantifying, and Correcting for Bias in Post-hoc Analysis of Gene-Based Rare Variant Tests of Association
Source: Front Genet. 2017 Sep 14;8:117. doi: 10.3389/fgene.2017.00117 (PMC5603735; doi:10.3389/fgene.2017.00117)
Supplement: Supplementary file 1 [file SupplementaryMaterial.docx]

Supplementary Material

Illustrating, Quantifying and Correcting for Bias in Post-Hoc Analysis of Gene-Based Rare Variant Tests of Association

**Kelsey Grinde^1^, Jaron Arbet^2^, Alden Green^3^, Michael O’Connell^2^, Alessandra Valcarcel^4^, Jason Westra^5,6^, Nathan Tintle^6,*^**

*** Correspondence:** Nathan Tintle: Nathan.Tintle@dordt.edu

# Supplementary Figures and Tables

## Supplementary Figures


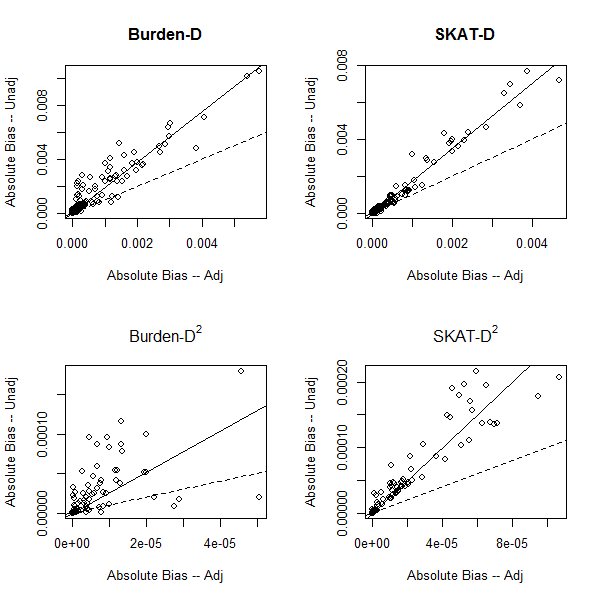
 **Supplementary Figure 1.** This figure shows the absolute value of the bias for the unadjusted statistic (y-axis) and adjusted statistic (x-axis) across all 50 simulation settings and all SNPs. The four panels of the figure represent the four combinations of stage 1 tests and stage 2 post-hoc statistics. Results are shown here for Stage 1 alpha level of 0.01. The dashed line represents the y=x line. Any points falling above this dashed line represent points at which our adjusted statistics have smaller bias than do the unadjusted statistics. Note that this happens almost all the time. Points falling below the dashed line are times when our adjusted statistic has larger bias than does the unadjusted statistic; this happens rarely, and when it does, the bias of our adjusted statistic is usually only very slightly larger than the bias of the unadjusted statistic (represented by the closeness of these points to the y=x line). The solid line is the line of best fit (constrained to have intercept 0). The slope of this line represents the average relative improvement of the bias of our adjusted statistics relative to the bias of our unadjusted statistics.


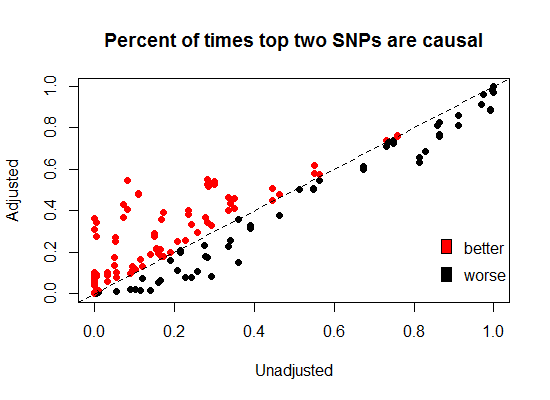


**Supplementary Figure 2.** This figure shows the percent of times both of the top two ranked SNPs are causal when ranking is based on the adjusted statistic versus the unadjusted statistic. Points are colored by whether or not the adjusted statistic provides “better” ranking results, where a “better” ranking result is one in which a higher percent of the time the top 2 SNPs are causal. This figure displays results only for the situations where we used a significance level of 0.01 for the step 1 gene-based test. Results are displayed for all 40 non-null simulation settings and all four combinations of step 1 test and step 2 statistic. The dashed line is the y=x line. When ranking based on the adjusted statistics is better, it is often considerably better (shown by the distance from the red points to the y=x line). The times that ranking based on the adjusted statistics is not better tend to be settings when the percent of times the top two SNPs are causal was already quite high before adjustment. The times that we are worse we are not much worse, as you can see that the black points are often quite close to the y=x line.

**
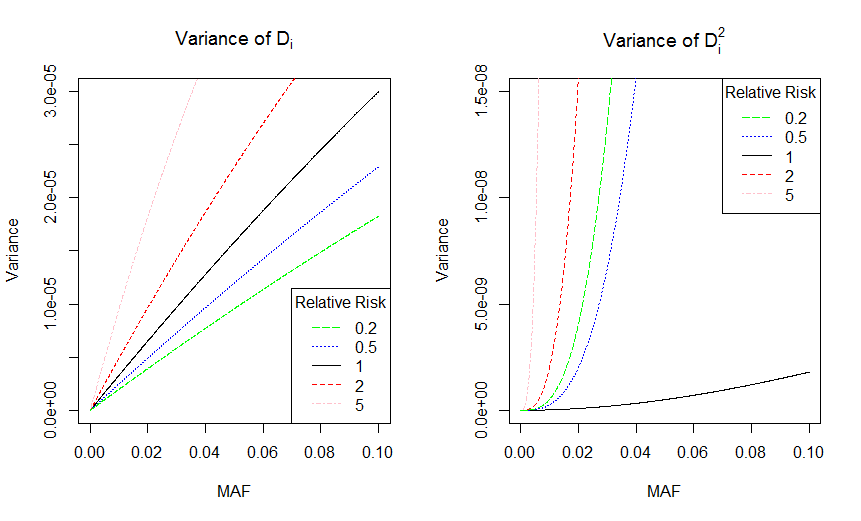
**

**Supplementary Figure 3.** Variance of unconditional single-marker post-hoc statistics $D_{i}$ and $D_{i}^{2}$ versus minor allele frequency in the cases ($f_{i}^{-}$) for different relative risks ($\lambda_{i}$).


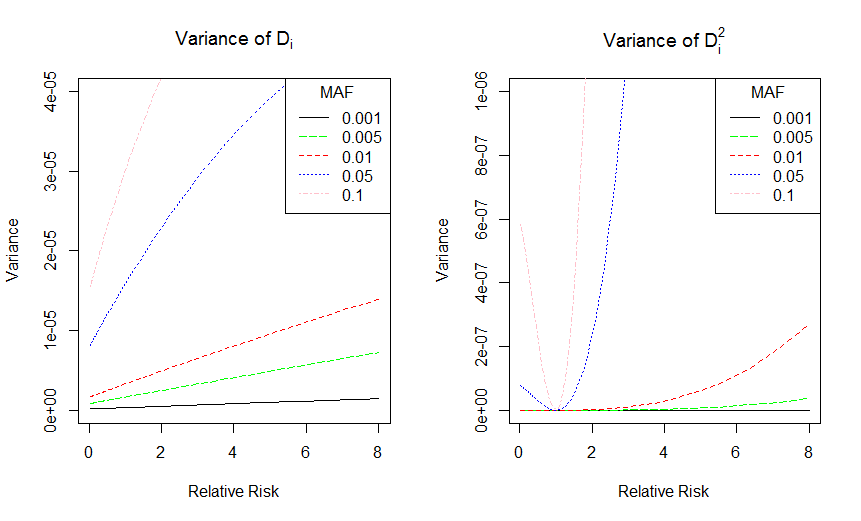


**Supplementary Figure 4.** Variance of unconditional single-marker post-hoc statistics versus relative risk for different minor allele frequencies.


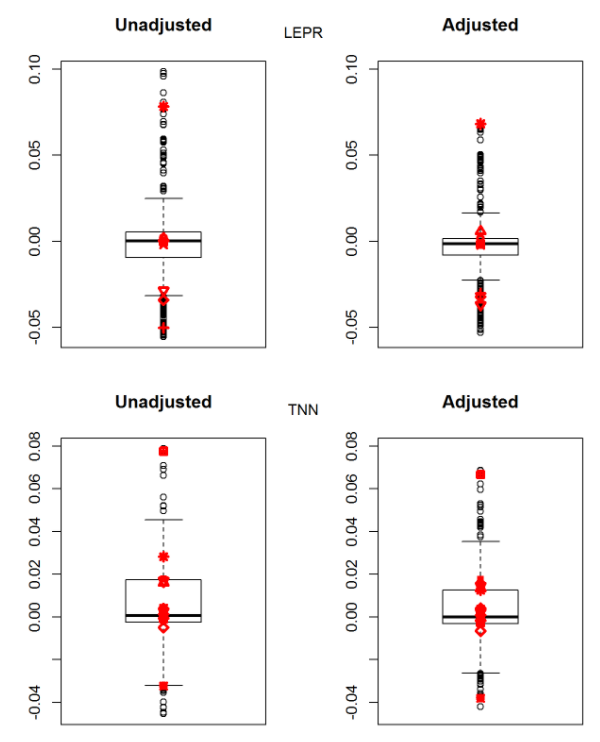


**Supplementary Figure 5.** Application of bootstrap resampling bias correction approach to real genetic data and simulated phenotypes from Genetic Analysis Workshop 18 (Bickeboller et al., 2014). Here we demonstrate the performance of our bootstrap resampling bias correction approach in two genes, LEPR and TNN, using statistics $Q_{bw}$at Step 1 and $D_{i}$ at Step 2. Panels **(A)-(D)** contain boxplots of post-hoc estimates of $\hat{D}_{i}$ for neutral variants, along with red points representing post-hoc estimates for the smaller number of causal variants. Panels on the left (**(A)**, **(C)**) show naïve (unadjusted) post-hoc estimates and panels on the right (**(B)**, **(D)**) show adjusted post-hoc estimates after applying our bias correction strategy. The top panels (**(A)**, **(B)**) show results for LEPR, and the bottom panels (**(C)**, **(D)**) show results for TNN. LEPR contains 8 causal variants and 935 neutral variants. Comparing panels **(A)** and **(B)**, we see that bias-corrected estimates of $\hat{D}_{i}$ for neutral variants have been shrunk toward their expected value of 0, as hoped. Also of note: the rank of the causal SNP with largest $D_{i}$ (the red asterisk) before adjustment was 13 (out of 943), but after adjustment the average rank was 1. TNN contains 15 causal variants and 514 neutral variants. Comparing panels **(C)** and **(D)**, we see that bias-corrected estimates of $\hat{D}_{i}$ for neutral variants have again been shrunk toward their expected value of 0. The most causal SNP in this gene (represented by the red square) maintains its rank as 5^th^ largest $\hat{D}_{i}$ after applying our bias correction strategy, but ends up just 2e-03 units away from the three variants in perfect LD that are tied at rank 1.

## Supplementary Tables

**Supplementary Table 1.** Simulation settings and Step 1 power (separate *.xls file, not contained here).

**Supplementary Table 2.** Overall improvement in bias and MSE of bias-adjusted statistics across 10,000 replications of the 40 alternative hypothesis simulation settings. Results shown for all four pairs of Step 1 and Step 2 test statistics.

| Step 1: GBT | Step 2: Single-marker statistic | Step 1 sig. level (%) | Bias or MSE^1^ | % of improved variants^2^ | Median improvement^3^ | Median decline^4^ | Median Increase/  Median Decrease^5^ |
| --- | --- | --- | --- | --- | --- | --- | --- |
| $Q_{bw}$ | $\hat{D}_{i}$ | 1 | Bias | 0.94 | 1.63x10^-04^ | 3.14x10^-05^ | 5.20 |
|  |  |  | MSE | 0.90 | 7.32x10^-08^ | 1.34x10^-08^ | 5.47 |
|  |  | 0.01 | Bias | 0.97 | 2.10x10^-04^ | 9.48x10^-05^ | 2.22 |
|  |  |  | MSE | 0.97 | 2.04x10^-07^ | 6.04x10^-08^ | 3.37 |
|  | $\hat{D}_{i}^{2}$ | 1 | Bias | 0.68 | 2.84x10^-07^ | 2.13x10^-08^ | 13.29 |
|  |  |  | MSE | 0.77 | 9.96x10^-13^ | 1.47x10^-14^ | 67.72 |
|  |  | 0.01 | Bias | 0.77 | 5.81x10^-07^ | 2.78x10^-08^ | 20.91 |
|  |  |  | MSE | 0.81 | 1.81x10^-12^ | 1.29x10^-14^ | 140.71 |
| $Q_{sw}$ | $\hat{D}_{i}$ | 1 | Bias | 0.77 | 6.31x10^-05^ | 6.10x10^-06^ | 10.35 |
|  |  |  | MSE | 0.92 | 1.15x10^-07^ | 1.92x10^-08^ | 5.98 |
|  |  | 0.01 | Bias | 0.74 | 9.91x10^-05^ | 2.31x10^-05^ | 4.29 |
|  |  |  | MSE | 0.88 | 1.66x10^-07^ | 2.90x10^-08^ | 5.72 |
|  | $\hat{D}_{i}^{2}$ | 1 | Bias | 0.64 | 2.37x10^-07^ | 2.06x10^-08^ | 11.51 |
|  |  |  | MSE | 0.77 | 1.11x10^-12^ | 1.68x10^-14^ | 65.95 |
|  |  | 0.01 | Bias | 0.69 | 4.85x10^-07^ | 2.81x10^-08^ | 17.29 |
|  |  |  | MSE | 0.81 | 2.85x10^-12^ | 1.92x10^-14^ | 148.22 |

^1^Bias is computed as the average difference between the estimated single-marker post-hoc statistic and its expected value. MSE is computed as the average squared difference between the estimated single-marker post-hoc statistic and its expected value.
^2^Computed as the percent of variants for which bias (or MSE, depending on the row of the table) decreased after implementing our bootstrap bias-correction strategy.
^3^The median change in bias (or MSE) among variants that show an improvement after adjustment (i.e., bias (or MSE) is smaller after adjustment).
^4^The median change in bias (or MSE) among variants that show a decline after adjustment (i.e., bias (or MSE) is larger after adjustment).
^5^The ratio of the previous two columns.

**Supplementary Table 3.** Overall improvement in bias and MSE of bias-adjusted statistics across 10,000 replications of the 10 null hypothesis simulation settings.

| Step 1: GBT | Step 2 Single-marker statistic | Step 1 sig. level | Bias or MSE | % of variants with reduced bias/MSE after adjustment | Median decrease in bias/MSE after adjustment | Median increase in bias/MSE | Median Decrease/ Median Increase |
| --- | --- | --- | --- | --- | --- | --- | --- |
| $Q_{bw}$ | $\hat{D}_{i}$ | 1% | Bias | 0.63 | 2.36x10^-5^ | 1.22x10^-5^ | 1.93 |
|  |  |  | MSE | 1.00 | 1.70x10^-7^ | - | - |
|  |  | 0.01% | Bias | 0.58 | 1.11x10^-4^ | 1.18x10^-4^ | 0.94 |
|  |  |  | MSE | 1.00 | 1.16x10^-7^ | - | - |
|  | $\hat{D}_{i}^{2}$ | 1% | Bias | 0.72 | 1.57x10^-7^ | 2.54x10^-8^ | 6.17 |
|  |  |  | MSE | 0.71 | 8.76x10^-13^ | 1.12x10^-14^ | 77.98 |
|  |  | 0.01% | Bias | 0.30 | 1.45x10^-6^ | 5.92x10^-8^ | 24.40 |
|  |  |  | MSE | 0.30 | 2.82x10^-12^ | 4.50x10^-15^ | 626.73 |
| $Q_{sw}$ | $\hat{D}_{i}$ | 1% | Bias | 0.61 | 1.64x10^-5^ | 5.11x10^-6^ | 3.22 |
|  |  |  | MSE | 1.00 | 1.08x10^-7^ | - | - |
|  |  | 0.01% | Bias | - | - | - | - |
|  |  |  | MSE | - | - | - | - |
|  | $\hat{D}_{i}^{2}$ | 1% | Bias | 0.53 | 1.08x10^-7^ | 2.02x10^-8^ | 5.37 |
|  |  |  | MSE | 0.63 | 8.70x10^-13^ | 1.86x10^-14^ | 46.69 |
|  |  | 0.01% | Bias | - | - | - | - |
|  |  |  | MSE | - | - | - | - |

**Supplementary Table 4.** Improvement in bias and MSE of bias-adjusted statistics stratified by the power of the Step 1 test. Results shown for all four pairs of Step 1 and Step 2 test statistics.

| Step 1: GBT | Step 2: Single-marker statistic | Step 1 sig. level (%) | Bias or MSE^1^ | Step 1 power | % of improved variants^2^ | Median improvement^3^ | Median decline^4^ | Median Increase/ Median Decrease^5^ |
| --- | --- | --- | --- | --- | --- | --- | --- | --- |
| $Q_{bw}$ | $\hat{D_{i}}$ | 1 | Bias | 0-0.05 | 0.90 | 1.77x10^-04^ | 3.24 x10^-05^ | 5.45 |
|  |  |  |  | 0.05-0.2 | 1.00 | 1.76 x10^-04^ | - | - |
|  |  |  |  | 0.2-0.5 | 1.00 | 1.52 x10^-04^ | - | - |
|  |  |  |  | 0.5-1 | 0.85 | 1.51 x10^-04^ | 1.67 x10^-05^ | 9.04 |
|  |  |  | MSE | 0-0.05 | 0.99 | 1.89 x10^-07^ | 1.07 x10^-09^ | 177 |
|  |  |  |  | 0.05-0.2 | 0.96 | 6.97 x10^-08^ | 1.13 x10^-08^ | 6.16 |
|  |  |  |  | 0.2-0.5 | 0.90 | 3.79 x10^-08^ | 1.28 x10^-08^ | 2.96 |
|  |  |  |  | 0.5-1 | 0.60 | 4.66 x10^-08^ | 3.52 x10^-08^ | 1.32 |
|  |  | 0.01 | Bias | 0-0.05 | 0.96 | 2.09 x10^-04^ | 9.48 x10^-05^ | 2.21 |
|  |  |  |  | 0.05-0.2 | 1.00 | 2.67 x10^-04^ | - | - |
|  |  |  |  | 0.2-0.5 | 1.00 | 1.83 x10^-04^ | - | - |
|  |  |  |  | 0.5-1 | - | - | - | - |
|  |  |  | MSE | 0-0.05 | 0.96 | 2.25 x10^-07^ | 6.04 x10^-08^ | 3.72 |
|  |  |  |  | 0.05-0.2 | 1.00 | 2.15 x10^-07^ | - | - |
|  |  |  |  | 0.2-0.5 | 1.00 | 5.10 x10^-08^ | - | - |
|  |  |  |  | 0.5-1 | - | - | - | - |
|  | $\hat{D_{i}^{2}}$ | 1 | Bias | 0-0.05 | 0.74 | 1.91 x10^-07^ | 2.60 x10^-08^ | 7.32 |
|  |  |  |  | 0.05-0.2 | 0.55 | 8.30 x10^-07^ | 1.77 x10^-08^ | 46.9 |
|  |  |  |  | 0.2-0.5 | 0.71 | 1.99 x10^-07^ | 5.14 x10^-08^ | 3.88 |
|  |  |  |  | 0.5-1 | 0.70 | 3.05 x10^-07^ | 2.19 x10^-08^ | 13.9 |
|  |  |  | MSE | 0-0.05 | 0.74 | 1.01 x10^-12^ | 1.02 x10^-14^ | 99.1 |
|  |  |  |  | 0.05-0.2 | 0.65 | 8.44 x10^-13^ | 1.35 x10^-14^ | 62.4 |
|  |  |  |  | 0.2-0.5 | 0.88 | 5.19 x10^-13^ | 1.63 x10^-14^ | 31.8 |
|  |  |  |  | 0.5-1 | 0.87 | 2.26 x10^-12^ | 2.03 x10^-14^ | 111 |
|  |  | 0.01 | Bias | 0-0.05 | 0.73 | 5.04 x10^-07^ | 2.78 x10^-08^ | 18.1 |
|  |  |  |  | 0.05-0.2 | 0.93 | 7.70 x10^-07^ | 2.54 x10^-08^ | 30.3 |
|  |  |  |  | 0.2-0.5 | 0.80 | 3.34 x10^-07^ | 1.98 x10^-08^ | 16.9 |
|  |  |  |  | 0.5-1 | - | - | - | - |
|  |  |  | MSE | 0-0.05 | 0.77 | 1.57 x10^-12^ | 1.26 x10^-14^ | 125 |
|  |  |  |  | 0.05-0.2 | 0.96 | 4.83 x10^-12^ | 1.40 x10^-14^ | 346 |
|  |  |  |  | 0.2-0.5 | 0.80 | 7.26 x10^-13^ | 1.68 x10^-14^ | 43.1 |
|  |  |  |  | 0.5-1 | - | - | - | - |
| $Q_{sw}$ | $\hat{D_{i}}$ | 1 | Bias | 0-0.05 | 0.73 | 6.19 x10^-05^ | 7.68 x10^-06^ | 8.06 |
|  |  |  |  | 0.05-0.2 | 0.81 | 5.48 x10^-05^ | 3.98 x10^-06^ | 13.8 |
|  |  |  |  | 0.2-0.5 | 0.80 | 6.70 x10^-05^ | 2.90 x10^-06^ | 23.1 |
|  |  |  |  | 0.5-1 | - | - | - | - |
|  |  |  | MSE | 0-0.05 | 0.96 | 1.09 x10^-07^ | 1.04 x10^-08^ | 10.4 |
|  |  |  |  | 0.05-0.2 | 0.85 | 1.45 x10^-07^ | 1.94 x10^-08^ | 7.48 |
|  |  |  |  | 0.2-0.5 | 0.97 | 7.05 x10^-08^ | 2.79 x10^-08^ | 2.52 |
|  |  |  |  | 0.5-1 | - | - | - | - |
|  |  | 0.01 | Bias | 0-0.05 | 0.74 | 9.91 x10^-05^ | 2.31 x10^-05^ | 4.29 |
|  |  |  |  | 0.05-0.2 | - | - | - | - |
|  |  |  |  | 0.2-0.5 | - | - | - | - |
|  |  |  |  | 0.5-1 | - | - | - | - |
|  |  |  | MSE | 0-0.05 | 0.88 | 1.66 x10^-07^ | 2.90 x10^-08^ | 5.72 |
|  |  |  |  | 0.05-0.2 | - | - | - | - |
|  |  |  |  | 0.2-0.5 | - | - | - | - |
|  |  |  |  | 0.5-1 | - | - | - | - |
|  | $\hat{D_{i}^{2}}$ | 1 | Bias | 0-0.05 | 0.68 | 1.49 x10^-07^ | 1.73 x10^-08^ | 8.60 |
|  |  |  |  | 0.05-0.2 | 0.62 | 4.75 x10^-07^ | 2.15 x10^-08^ | 22.1 |
|  |  |  |  | 0.2-0.5 | 0.55 | 2.43 x10^-07^ | 2.06 x10^-08^ | 11.8 |
|  |  |  |  | 0.5-1 | - | - | - | - |
|  |  |  | MSE | 0-0.05 | 0.74 | 9.76 x10^-13^ | 1.37 x10^-14^ | 71.4 |
|  |  |  |  | 0.05-0.2 | 0.81 | 2.45 x10^-12^ | 1.87 x10^-14^ | 131 |
|  |  |  |  | 0.2-0.5 | 0.77 | 1.52 x10^-12^ | 1.85 x10^-14^ | 82.4 |
|  |  |  |  | 0.5-1 | - | - | - | - |
|  |  | 0.01 | Bias | 0-0.05 | 0.69 | 4.85 x10^-07^ | 2.81 x10^-08^ | 17.3 |
|  |  |  |  | 0.05-0.2 | - | - | - | - |
|  |  |  |  | 0.2-0.5 | - | - | - | - |
|  |  |  |  | 0.5-1 | - | - | - | - |
|  |  |  | MSE | 0-0.05 | 0.81 | 2.85 x10^-12^ | 1.92 x10^-14^ | 148 |
|  |  |  |  | 0.05-0.2 | - | - | - | - |
|  |  |  |  | 0.2-0.5 | - | - | - | - |
|  |  |  |  | 0.5-1 | - | - | - | - |

^1^Bias is computed as the average difference between the estimated single-marker post-hoc statistic and its expected value. MSE is computed as the average squared difference between the estimated single-marker post-hoc statistic and its expected value.

^2^Computed as the percent of variants for which bias (or MSE, depending on the row of the table) decreased after implementing our bootstrap bias-correction strategy. Set to - if no step 1 tests had power in that range.
^3^The median change in bias (or MSE) among variants that show an improvement after adjustment (i.e., bias (or MSE) is smaller after adjustment). Set to - if no tests in that range.
^4^The median change in bias (or MSE) among variants that show a decline after adjustment (i.e., bias (or MSE) is larger after adjustment). Set to - if no tests in that range or no variants showed decline (i.e., % of improved variants is 1).
^5^The ratio of the previous two columns. Set to - if either of previous two columns is -.

**Supplementary Table 5.** Improvement in bias and MSE of bias-adjusted statistics stratified by the MAF and relative risk of the variant.

| Step 1: GBT | Step 2: Single-marker statistic |  | Step 1 sig. level | Bias or MSE | MAF | Rel. Risk | % of improved variants | Median improvement | Median decline | Median Decrease/ Median Increase |
| --- | --- | --- | --- | --- | --- | --- | --- | --- | --- | --- |
| $Q_{bw}$ | $\hat{D}_{i}$ |  | 1% | Bias | 1.00E-04 | 0.125 | 0.25 | 2.85 x10^-05^ | 3.24 x10^-05^ | 0.88 |
|  |  |  |  |  | 1.00E-04 | 1 | 0.99 | 3.21 x10^-05^ | 2.66 x10^-06^ | 1.20 |
|  |  |  |  |  | 1.00E-04 | 8 | 0.91 | 1.11 x10^-04^ | 1.46 x10^-05^ | 7.60 |
|  |  |  |  |  | 0.001 | 0.5 | 0.88 | 1.03 x10^-04^ | 4.33 x10^-05^ | 2.37 |
|  |  |  |  |  | 0.001 | 1 | 0.98 | 1.88 x10^-04^ | 2.08 x10^-05^ | 9.07 |
|  |  |  |  |  | 0.001 | 2 | 0.99 | 2.54 x10^-04^ | 2.48 x10^-05^ | 10.2 |
|  |  |  |  |  | 0.01 | 0.67 | 1.00 | 9.14 x10^-04^ | - | - |
|  |  |  |  |  | 0.01 | 1 | 1.00 | 1.33 x10^-03^ | - | - |
|  |  |  |  |  | 0.01 | 1.5 | 1.00 | 1.58 x10^-03^ | - | - |
|  |  |  |  |  | 0.05 | 0.83 | 0.50 | 9.47 x10^-04^ | 2.30 x10^-04^ | 4.11 |
|  |  |  |  |  | 0.05 | 1 | 0.94 | 2.63 x10^-03^ | 7.49 x10^-05^ | 35.1 |
|  |  |  |  |  | 0.05 | 1.2 | 0.63 | 2.49 x10^-03^ | 3.42 x10^-05^ | 72.7 |
|  |  |  |  | MSE | 1.00E-04 | 0.125 | 0.63 | 5.53 x10^-09^ | 1.80 x10^-08^ | 30.7 |
|  |  |  |  |  | 1.00E-04 | 1 | 0.97 | 2.36 x10^-08^ | 1.81 x10^-09^ | 13.0 |
|  |  |  |  |  | 1.00E-04 | 8 | 0.71 | 1.95 x10^-08^ | 1.38 x10^-08^ | 1.42 |
|  |  |  |  |  | 0.001 | 0.5 | 1.00 | 1.01 x10^-07^ | - | - |
|  |  |  |  |  | 0.001 | 1 | 0.96 | 1.57 x10^-07^ | 5.33 x10^-09^ | 29.5 |
|  |  |  |  |  | 0.001 | 2 | 1.00 | 1.21 x10^-07^ | - | - |
|  |  |  |  |  | 0.01 | 0.67 | 1.00 | 4.81 x10^-06^ | - | - |
|  |  |  |  |  | 0.01 | 1 | 0.94 | 5.90 x10^-06^ | 1.00 x10^-08^ | 587 |
|  |  |  |  |  | 0.01 | 1.5 | 0.75 | 6.00 x10^-06^ | 4.04 x10^-07^ | 15 |
|  |  |  |  |  | 0.05 | 0.83 | 1.00 | 3.52 x10^-05^ | - | - |
|  |  |  |  |  | 0.05 | 1 | 0.88 | 2.93 x10^-05^ | 1.41 x10^-06^ | 20.7 |
|  |  |  |  |  | 0.05 | 1.2 | 0.75 | 1.43 x10^-05^ | 5.69 x10^-06^ | 2.51 |
|  |  |  | 0.01% | Bias | 1.00E-04 | 0.125 | 0.75 | - | 4.36 x10^-05^ | - |
|  |  |  |  |  | 1.00E-04 | 1 | 0.94 | 5.63 x10^-05^ | 4.32 x10^-05^ | 1.30 |
|  |  |  |  |  | 1.00E-04 | 8 | 1.00 | 1.56 x10^-04^ | - | - |
|  |  |  |  |  | 0.001 | 0.5 | 0.50 | 1.67 x10^-04^ | 1.10 x10^-04^ | 1.51 |
|  |  |  |  |  | 0.001 | 1 | 0.93 | 2.09 x10^-04^ | 9.75 x10^-05^ | 2.15 |
|  |  |  |  |  | 0.001 | 2 | 1.00 | 3.28 x10^-04^ | - | - |
|  |  |  |  |  | 0.01 | 0.67 | 1.00 | 1.62 x10^-03^ | - | - |
|  |  |  |  |  | 0.01 | 1 | 1.00 | 1.68 x10^-03^ | - | - |
|  |  |  |  |  | 0.01 | 1.5 | 1.00 | 1.94 x10^-03^ | - | - |
|  |  |  |  |  | 0.05 | 0.83 | 1.00 | 2.33 x10^-03^ | - | - |
|  |  |  |  |  | 0.05 | 1 | 1.00 | 3.53 x10^-03^ | - | - |
|  |  |  |  |  | 0.05 | 1.2 | 1.00 | 3.17 x10^-03^ | - | - |
|  |  |  |  | MSE | 1.00E-04 | 0.125 | 0.75 | 3.70 x10^-08^ | 6.20 x10^-08^ | 0.60 |
|  |  |  |  |  | 1.00E-04 | 1 | 0.93 | 2.78 x10^-08^ | 2.18 x10^-08^ | 1.28 |
|  |  |  |  |  | 1.00E-04 | 8 | 0.99 | 7.93 x10^-08^ | 4.76 x10^-09^ | 16.6 |
|  |  |  |  |  | 0.001 | 0.5 | 0.50 | 2.22 x10^-07^ | 2.83 x10^-07^ | 0.79 |
|  |  |  |  |  | 0.001 | 1 | 0.97 | 2.16 x10^-07^ | 1.47 x10^-07^ | 1.47 |
|  |  |  |  |  | 0.001 | 2 | 0.99 | 3.34 x10^-07^ | 6.04 x10^-08^ | 5.54 |
|  |  |  |  |  | 0.01 | 0.67 | 1.00 | 6.00 x10^-06^ | - | - |
|  |  |  |  |  | 0.01 | 1 | 1.00 | 1.30 x10^-05^ | - | - |
|  |  |  |  |  | 0.01 | 1.5 | 1.00 | 1.11 x10^-05^ | - | - |
|  |  |  |  |  | 0.05 | 0.83 | 1.00 | 3.95 x10^-05^ | - | - |
|  |  |  |  |  | 0.05 | 1 | 1.00 | 4.84 x10^-05^ | - | - |
|  |  |  |  |  | 0.05 | 1.2 | 1.00 | 2.97 x10^-05^ | - | - |
|  | $\hat{D}_{i}^{2}$ |  | 1% | Bias | 1.00E-04 | 0.125 | 0.25 | 1.08 x10^-08^ | 1.07 x10^-08^ | 1.01 |
|  |  |  |  |  | 1.00E-04 | 1 | 0.12 | 1.76 x10^-08^ | 2.06 x10^-08^ | 0.85 |
|  |  |  |  |  | 1.00E-04 | 8 | 0.97 | 1.72 x10^-07^ | 1.29 x10^-08^ | 13.3 |
|  |  |  |  |  | 0.001 | 0.5 | 0.75 | 4.89 x10^-08^ | 2.25 x10^-08^ | 2.18 |
|  |  |  |  |  | 0.001 | 1 | 0.67 | 1.36 x10^-07^ | 2.02 x10^-08^ | 6.73 |
|  |  |  |  |  | 0.001 | 2 | 1.00 | 5.57 x10^-07^ | - | - |
|  |  |  |  |  | 0.01 | 0.67 | 0.50 | 8.70 x10^-07^ | 6.82 x10^-07^ | 1.28 |
|  |  |  |  |  | 0.01 | 1 | 0.81 | 7.28 x10^-06^ | 1.85 x10^-07^ | 39.4 |
|  |  |  |  |  | 0.01 | 1.5 | 1.00 | 2.32 x10^-05^ | - | - |
|  |  |  |  |  | 0.05 | 0.83 | 0.00 | - | 1.70 x10^-05^ | - |
|  |  |  |  |  | 0.05 | 1 | 0.75 | 2.81 x10^-05^ | 1.95 x10^-06^ | 14.4 |
|  |  |  |  |  | 0.05 | 1.2 | 0.75 | 5.30 x10^-05^ | 1.60 x10^-05^ | 3.32 |
|  |  |  |  | MSE | 1.00E-04 | 0.125 | 0.00 | - | 1.16 x10^-14^ | - |
|  |  |  |  |  | 1.00E-04 | 1 | 0.04 | 3.48 x10^-15^ | 1.47 x10^-14^ | 0.24 |
|  |  |  |  |  | 1.00E-04 | 8 | 1.00 | 4.86 x10^-13^ | - | - |
|  |  |  |  |  | 0.001 | 0.5 | 1.00 | 2.84 x10^-13^ | - | - |
|  |  |  |  |  | 0.001 | 1 | 1.00 | 5.67 x10^-13^ | - | - |
|  |  |  |  |  | 0.001 | 2 | 1.00 | 3.40 x10^-12^ | - | - |
|  |  |  |  |  | 0.01 | 0.67 | 1.00 | 9.24 x10^-11^ | - | - |
|  |  |  |  |  | 0.01 | 1 | 1.00 | 1.18 x10^-10^ | - | - |
|  |  |  |  |  | 0.01 | 1.5 | 1.00 | 9.44 x10^-10^ | - | - |
|  |  |  |  |  | 0.05 | 0.83 | 1.00 | 9.02 x10^-09^ | - | - |
|  |  |  |  |  | 0.05 | 1 | 1.00 | 1.86 x10^-09^ | - | - |
|  |  |  |  |  | 0.05 | 1.2 | 1.00 | 8.35 x10^-09^ | - | - |
|  |  |  | 0.01% | Bias | 1.00E-04 | 0.125 | 0.50 | - | 5.33 x10^-08^ | - |
|  |  |  |  |  | 1.00E-04 | 1 | 0.21 | 1.34 x10^-08^ | 2.60 x10^-08^ | 0.51 |
|  |  |  |  |  | 1.00E-04 | 8 | 0.99 | 3.10 x10^-07^ | 2.74 x10^-08^ | 11.3 |
|  |  |  |  |  | 0.001 | 0.5 | 0.50 | 3.49 x10^-07^ | 5.56 x10^-08^ | 6.29 |
|  |  |  |  |  | 0.001 | 1 | 0.82 | 1.72 x10^-07^ | 5.19 x10^-08^ | 3.31 |
|  |  |  |  |  | 0.001 | 2 | 0.97 | 9.87 x10^-07^ | 2.34 x10^-07^ | 4.21 |
|  |  |  |  |  | 0.01 | 0.67 | 1.00 | 2.02 x10^-06^ | - | - |
|  |  |  |  |  | 0.01 | 1 | 1.00 | 1.47 x10^-05^ | - | - |
|  |  |  |  |  | 0.01 | 1.5 | 1.00 | 3.04 x10^-05^ | - | - |
|  |  |  |  |  | 0.05 | 0.83 | 1.00 | 1.38 x10^-06^ | - | - |
|  |  |  |  |  | 0.05 | 1 | 1.00 | 5.19 x10^-05^ | - | - |
|  |  |  |  |  | 0.05 | 1.2 | 1.00 | 1.04 x10^-04^ | - | - |
|  |  |  |  | MSE | 1.00E-04 | 0.125 | 0.75 | 7.71 x10^-16^ | 8.47 x10^-14^ | 9.10^-03^ |
|  |  |  |  |  | 1.00E-04 | 1 | 0.21 | 6.74 x10^-15^ | 1.22 x10^-14^ | 0.55 |
|  |  |  |  |  | 1.00E-04 | 8 | 0.99 | 8.18 x10^-13^ | 1.96 x10^-14^ | 41.7 |
|  |  |  |  |  | 0.001 | 0.5 | 0.50 | 2.09 x10^-13^ | 7.71 x10^-14^ | 2.71 |
|  |  |  |  |  | 0.001 | 1 | 0.95 | 6.26 x10^-13^ | 8.89 x10^-14^ | 7.05 |
|  |  |  |  |  | 0.001 | 2 | 1.00 | 6.49 x10^-12^ | - | - |
|  |  |  |  |  | 0.01 | 0.67 | 1.00 | 5.89 x10^-11^ | - | - |
|  |  |  |  |  | 0.01 | 1 | 1.00 | 4.54 x10^-10^ | - | - |
|  |  |  |  |  | 0.01 | 1.5 | 1.00 | 3.13 x10^-09^ | - | - |
|  |  |  |  |  | 0.05 | 0.83 | 1.00 | 2.44 x10^-10^ | - | - |
|  |  |  |  |  | 0.05 | 1 | 1.00 | 6.58 x10^-09^ | - | - |
|  |  |  |  |  | 0.05 | 1.2 | 1.00 | 2.82 x10^-08^ | - | - |
| $Q_{sw}$ | $\hat{D}_{i}$ |  | 1% | Bias | 1.00E-04 | 0.125 | 0.00 | - | 5.53 x10^-05^ | - |
|  |  |  |  |  | 1.00E-04 | 1 | 0.60 | 3.69 x10^-06^ | 4.77 x10^-06^ | 0.77 |
|  |  |  |  |  | 1.00E-04 | 8 | 0.82 | 7.49 x10^-05^ | 3.19 x10^-05^ | 2.35 |
|  |  |  |  |  | 0.001 | 0.5 | 1.00 | 4.67 x10^-05^ | - | - |
|  |  |  |  |  | 0.001 | 1 | 0.66 | 6.03 x10^-06^ | 4.80 x10^-06^ | 1.26 |
|  |  |  |  |  | 0.001 | 2 | 1.00 | 1.32 x10^-04^ | - | - |
|  |  |  |  |  | 0.01 | 0.67 | 1.00 | 7.75 x10^-04^ | - | - |
|  |  |  |  |  | 0.01 | 1 | 0.75 | 4.63 x10^-05^ | 4.16 x10^-05^ | 1.11 |
|  |  |  |  |  | 0.01 | 1.5 | 1.00 | 1.56 x10^-03^ | - | - |
|  |  |  |  |  | 0.05 | 0.83 | 1.00 | 2.23 x10^-03^ | - | - |
|  |  |  |  |  | 0.05 | 1 | 0.94 | 8.62 x10^-05^ | 4.91 x10^-06^ | 17.6 |
|  |  |  |  |  | 0.05 | 1.2 | 1.00 | 2.37 x10^-03^ | - | - |
|  |  |  |  | MSE | 1.00E-04 | 0.125 | 0.88 | 7.35 x10^-09^ | 3.16 x10^-10^ | 23.3 |
|  |  |  |  |  | 1.00E-04 | 1 | 1.00 | 2.30 x10^-08^ | - | - |
|  |  |  |  |  | 1.00E-04 | 8 | 0.58 | 5.04 x10^-08^ | 1.93 x10^-08^ | 2.62 |
|  |  |  |  |  | 0.001 | 0.5 | 1.00 | 5.84 x10^-08^ | - | - |
|  |  |  |  |  | 0.001 | 1 | 1.00 | 1.09 x10^-07^ | - | - |
|  |  |  |  |  | 0.001 | 2 | 1.00 | 3.24 x10^-07^ | - | - |
|  |  |  |  |  | 0.01 | 0.67 | 1.00 | 2.80 x10^-06^ | - | - |
|  |  |  |  |  | 0.01 | 1 | 1.00 | 8.05 x10^-06^ | - | - |
|  |  |  |  |  | 0.01 | 1.5 | 1.00 | 7.59 x10^-06^ | - | - |
|  |  |  |  |  | 0.05 | 0.83 | 1.00 | 1.46 x10^-05^ | - | - |
|  |  |  |  |  | 0.05 | 1 | 1.00 | 2.71 x10^-05^ | - | - |
|  |  |  |  |  | 0.05 | 1.2 | 1.00 | 2.28 x10^-05^ | - | - |
|  |  |  | 0.01% | Bias | 1.00E-04 | 0.125 | 0.50 | - | 4.52 x10^-05^ | - |
|  |  |  |  |  | 1.00E-04 | 1 | 0.66 | 2.17 x10^-05^ | 1.31 x10^-05^ | 1.65 |
|  |  |  |  |  | 1.00E-04 | 8 | 0.74 | 5.60 x10^-05^ | 4.29 x10^-05^ | 1.31 |
|  |  |  |  |  | 0.001 | 0.5 | 1.00 | 7.78 x10^-05^ | - | - |
|  |  |  |  |  | 0.001 | 1 | 0.61 | 1.42 x10^-05^ | 1.22 x10^-05^ | 1.17 |
|  |  |  |  |  | 0.001 | 2 | 0.85 | 1.56 x10^-04^ | 4.48 x10^-05^ | 3.48 |
|  |  |  |  |  | 0.01 | 0.67 | 1.00 | 1.04 x10^-03^ | - | - |
|  |  |  |  |  | 0.01 | 1 | 0.80 | 6.80 x10^-05^ | 3.40 x10^-04^ | 0.20 |
|  |  |  |  |  | 0.01 | 1.5 | 1.00 | 2.19 x10^-03^ | - | - |
|  |  |  |  |  | 0.05 | 0.83 | 1.00 | 3.37 x10^-03^ | - | - |
|  |  |  |  |  | 0.05 | 1 | 0.64 | 2.89 x10^-04^ | 9.12 x10^-05^ | 3.17 |
|  |  |  |  |  | 0.05 | 1.2 | 1.00 | 3.56 x10^-03^ | - | - |
|  |  |  |  | MSE | 1.00E-04 | 0.125 | 0.50 | - | 2.43 x10^-09^ | - |
|  |  |  |  |  | 1.00E-04 | 1 | 0.96 | 2.24 x10^-08^ | 7.36 x10^-09^ | 3.04 |
|  |  |  |  |  | 1.00E-04 | 8 | 0.47 | 1.81 x10^-07^ | 3.27 x10^-08^ | 5.52 |
|  |  |  |  |  | 0.001 | 0.5 | 1.00 | 1.11 x10^-07^ | - | - |
|  |  |  |  |  | 0.001 | 1 | 0.95 | 9.28 x10^-08^ | 1.29 x10^-07^ | 0.72 |
|  |  |  |  |  | 0.001 | 2 | 0.95 | 4.05 x10^-07^ | 4.21 x10^-08^ | 9.64 |
|  |  |  |  |  | 0.01 | 0.67 | 1.00 | 6.48 x10^-06^ | - | - |
|  |  |  |  |  | 0.01 | 1 | 1.00 | 5.70 x10^-06^ | - | - |
|  |  |  |  |  | 0.01 | 1.5 | 1.00 | 2.46 x10^-05^ | - | - |
|  |  |  |  |  | 0.05 | 0.83 | 1.00 | 5.22 x10^-05^ | - | - |
|  |  |  |  |  | 0.05 | 1 | 1.00 | 3.80 x10^-05^ | - | - |
|  |  |  |  |  | 0.05 | 1.2 | 1.00 | 7.51 x10^-05^ | - | - |
|  | $\hat{D}_{i}^{2}$ |  | 1% | Bias | 1.00E-04 | 0.125 | 0.13 | 2.81 x10^-09^ | 1.75 x10^-08^ | 0.16 |
|  |  |  |  |  | 1.00E-04 | 1 | 0.11 | 6.56 x10^-09^ | 1.78 x10^-08^ | 0.37 |
|  |  |  |  |  | 1.00E-04 | 8 | 0.92 | 1.14 x10^-07^ | 3.58 x10^-08^ | 3.19 |
|  |  |  |  |  | 0.001 | 0.5 | 0.75 | 9.68 x10^-08^ | 7.72 x10^-09^ | 12.5 |
|  |  |  |  |  | 0.001 | 1 | 0.44 | 4.94 x10^-08^ | 4.44 x10^-08^ | 1.11 |
|  |  |  |  |  | 0.001 | 2 | 1.00 | 5.15 x10^-07^ | - | - |
|  |  |  |  |  | 0.01 | 0.67 | 1.00 | 8.35 x10^-06^ | - | - |
|  |  |  |  |  | 0.01 | 1 | 1.00 | 8.45 x10^-06^ | - | - |
|  |  |  |  |  | 0.01 | 1.5 | 1.00 | 2.52 x10^-05^ | - | - |
|  |  |  |  |  | 0.05 | 0.83 | 1.00 | 5.72 x10^-05^ | - | - |
|  |  |  |  |  | 0.05 | 1 | 1.00 | 2.76 x10^-05^ | - | - |
|  |  |  |  |  | 0.05 | 1.2 | 1.00 | 7.49 x10^-05^ | - | - |
|  |  |  |  | MSE | 1.00E-04 | 0.125 | 0.13 | 7.17 x10^-15^ | 1.03 x10^-14^ | 0.70 |
|  |  |  |  |  | 1.00E-04 | 1 | 0.04 | 2.95 x10^-15^ | 1.77 x10^-14^ | 0.17 |
|  |  |  |  |  | 1.00E-04 | 8 | 1.00 | 5.40 x10^-13^ | - | - |
|  |  |  |  |  | 0.001 | 0.5 | 1.00 | 5.26 x10^-13^ | - | - |
|  |  |  |  |  | 0.001 | 1 | 1.00 | 4.70 x10^-13^ | - | - |
|  |  |  |  |  | 0.001 | 2 | 1.00 | 6.57 x10^-12^ | - | - |
|  |  |  |  |  | 0.01 | 0.67 | 1.00 | 3.09 x10^-10^ | - | - |
|  |  |  |  |  | 0.01 | 1 | 1.00 | 3.39 x10^-10^ | - | - |
|  |  |  |  |  | 0.01 | 1.5 | 1.00 | 1.34 x10^-09^ | - | - |
|  |  |  |  |  | 0.05 | 0.83 | 1.00 | 8.01 x10^-09^ | - | - |
|  |  |  |  |  | 0.05 | 1 | 1.00 | 4.23 x10^-09^ | - | - |
|  |  |  |  |  | 0.05 | 1.2 | 1.00 | 1.62 x10^-08^ | - | - |
|  |  |  | 0.01% | Bias | 1.00E-04 | 0.125 | 0.50 | - | 5.45 x10^-09^ | - |
|  |  |  |  |  | 1.00E-04 | 1 | 0.31 | 1.25 x10^-08^ | 2.22 x10^-08^ | 0.57 |
|  |  |  |  |  | 1.00E-04 | 8 | 0.74 | 7.78 x10^-08^ | 1.73 x10^-08^ | 4.50 |
|  |  |  |  |  | 0.001 | 0.5 | 1.00 | 3.58 x10^-08^ | - | - |
|  |  |  |  |  | 0.001 | 1 | 0.50 | 9.17 x10^-08^ | 5.20 x10^-08^ | 1.76 |
|  |  |  |  |  | 0.001 | 2 | 0.92 | 6.49 x10^-07^ | 4.79 x10^-08^ | 13.5 |
|  |  |  |  |  | 0.01 | 0.67 | 1.00 | 1.43 x10^-05^ | - | - |
|  |  |  |  |  | 0.01 | 1 | 1.00 | 5.53 x10^-06^ | - | - |
|  |  |  |  |  | 0.01 | 1.5 | 1.00 | 5.10 x10^-05^ | - | - |
|  |  |  |  |  | 0.05 | 0.83 | 1.00 | 1.20 x10^-04^ | - | - |
|  |  |  |  |  | 0.05 | 1 | 1.00 | 3.93 x10^-05^ | - | - |
|  |  |  |  |  | 0.05 | 1.2 | 1.00 | 1.58 x10^-04^ | - | - |
|  |  |  |  | MSE | 1.00E-04 | 0.125 | 0.50 | - | 1.78 x10^-14^ | - |
|  |  |  |  |  | 1.00E-04 | 1 | 0.10 | 8.49 x10^-14^ | 1.87 x10^-14^ | 4.54 |
|  |  |  |  |  | 1.00E-04 | 8 | 0.97 | 3.31 x10^-13^ | 8.16 x10^-14^ | 4.06 |
|  |  |  |  |  | 0.001 | 0.5 | 1.00 | 4.31 x10^-13^ | - | - |
|  |  |  |  |  | 0.001 | 1 | 0.97 | 3.93 x10^-13^ | 5.35 x10^-14^ | 7.34 |
|  |  |  |  |  | 0.001 | 2 | 0.97 | 7.69 x10^-12^ | 5.17 x10^-14^ | 149 |
|  |  |  |  |  | 0.01 | 0.67 | 1.00 | 1.08 x10^-09^ | - | - |
|  |  |  |  |  | 0.01 | 1 | 1.00 | 3.11 x10^-10^ | - | - |
|  |  |  |  |  | 0.01 | 1.5 | 1.00 | 8.29 x10^-09^ | - | - |
|  |  |  |  |  | 0.05 | 0.83 | 1.00 | 4.90 x10^-08^ | - | - |
|  |  |  |  |  | 0.05 | 1 | 1.00 | 1.07 x10^-08^ | - | - |
|  |  |  |  |  | 0.05 | 1.2 | 1.00 | 1.01 x10^-07^ | - | - |
