# Supplementary material for: Illustrating, Quantifying, and Correcting for Bias in Post-hoc Analysis of Gene-Based Rare Variant Tests of Association
Source: Front Genet. 2017 Sep 14;8:117. doi: 10.3389/fgene.2017.00117 (PMC5603735; doi:10.3389/fgene.2017.00117)
Supplement: Supplementary file 3 [file Appendix.docx]

**Appendix. Intuition for behavior of single-marker statistics after significant Step 1 gene-based test**

Here we provide a detailed demonstration of the behavior of particular Step 2 single-marker statistics following a significant Step 1 gene-based test (GBT).

**A.1 Distribution of post-hoc single-marker test statistics**

Consider a collection of single-marker test statistics $T_{i}$ measuring the association between the genotype at marker $i$ and some phenotype of interest. Suppose we perform a Step 1 gene-based test to test the association between the gene containing markers $i= 1,\ldots,m$and the phenotype using a test statistic of the form$Q= \sum_{i=1}^{m} w_{i}T_{i}$, for some weights $w_{i}$ (e.g., burden test with $T_{i}=D_{i}$, variance components test with $T_{i}=D_{i}^{2}$).

Assume that these single-marker statistics are asymptotically multivariate normal with known mean and variance: $\boldsymbol{T}= \left[ \begin{matrix} T_{1} & \ldots& T_{m} \end{matrix} \right]^{T}\sim N_{m}(\boldsymbol{\mu},\boldsymbol{\Sigma})$. (See Conneely and Boehnke (2007) for verification of the validity of this assumption---in the presence of arbitrary LD structure and minor allele frequencies, for continuous or dichotomous traits, and with or without covariates---for many commonly-used single-marker tests of genotype-phenotype association.)

The vector $\left[ \begin{matrix} T_{i} \\ Q \end{matrix} \right]$ can be re-written as $\boldsymbol{AT}$**,** for $\boldsymbol{A}=\left[ \begin{matrix} \delta_{i1} & \ldots& \delta_{im} \\ w_{1} & \ldots& w_{m} \end{matrix} \right]$, where $\delta_{ij}$ is the Kronecker delta. It follows that the joint distribution of $T_{i}$ and $Q$ is

$$\left[ \begin{matrix} T_{i} \\ Q \end{matrix} \right]\sim N\left( \boldsymbol{A\mu}, \boldsymbol{A}\boldsymbol{\Sigma}\boldsymbol{A}^{\boldsymbol{T}} \right)$$

$$\equiv N\left( \left[ \begin{matrix} \mu_{i} \\ \sum_{j=1}^{m} w_{j}\mu_{j} \end{matrix} \right], \left[ \begin{matrix} \sigma_{ii} & \sum_{j=1}^{m} w_{j}\sigma_{ij} \\ \sum_{j=1}^{m} w_{j}\sigma_{ji} & \sum_{j=1}^{m} \sum_{k=1}^{m} w_{j}{w_{k}\sigma}_{jk} \end{matrix} \right] \right).$$

Then, the conditional distribution of $T_{i}$ given $Q$ is

$$T_{i}|Q\sim N\left( \mu_{i}+\frac{\sum_{j} w_{j}\sigma_{ij}}{\sum_{j} \sum_{k} w_{j}w_{k}\sigma_{jk}}\left( Q-\sum_{j} w_{j}\mu_{j} \right), \sigma_{ii}-\frac{\left( \sum_{j} w_{j}\sigma_{ij} \right)^{2}}{\sum_{j} \sum_{k} w_{j}w_{k}\sigma_{jk}} \right)$$

$$\equiv N\left( \mu_{i}+\frac{w_{i}\sigma_{ii}+\sum_{j\neq i} w_{j}\sigma_{ij}}{V\left( Q \right)}\left( Q-\sum_{j} w_{j}\mu_{j} \right), \sigma_{ii}-\frac{\left( w_{i}\sigma_{ii}+\sum_{j\neq i} w_{j}\sigma_{ij} \right)^{2}}{V\left( Q \right)} \right)$$

$$\equiv N\left( \mu_{i}+\frac{w_{i}V\left( T_{i} \right)+\sum_{j\neq i} w_{j}\sigma_{ij}}{V\left( Q \right)}\mathrm{Bias}\left( Q \right), V\left( T_{i} \right)-\frac{\left( w_{i}V\left( T_{i} \right)+\sum_{j\neq i} w_{j}\sigma_{ij} \right)^{2}}{V\left( Q \right)} \right).$$

**A.1.1 Post-hoc bias**

From the form of the conditional distribution of the single-marker statistic $T_{i}$ given the GBT $Q$, it follows that the form of the post-hoc bias (i.e., the bias of $T_{i}$ given $Q$) is:

$$\mathrm{Bias}\left( T_{i} \right|Q)=E\left( T_{i} \right|Q)- \mu_{i} = \mu_{i}+\frac{w_{i}V\left( T_{i} \right)+\sum_{j\neq i} w_{j}\sigma_{ij}}{V\left( Q \right)}\mathrm{Bias}\left( Q \right)- \mu_{i} =\frac{w_{i}V\left( T_{i} \right)+\sum_{j\neq i} w_{j}\sigma_{ij}}{V\left( Q \right)}\mathrm{Bias}\left( Q \right).$$

We can see from this equation that the post-hoc bias will increase as:

1. The bias of the GBT statistic increases. (Note: if we only compute single-marker statistics after significant Step 1 tests, then $Q$ is necessarily very large and $\mathrm{Bias}(Q)$ will also be large due to winner’s curse, particularly if the GBT $Q$ is not well powered.)
2. The variance of the GBT statistic decreases.
3. The variance of the single-marker statistic increases. (Note: for many test statistics $V(T_{i})$ increases with MAF (see Section A.2 for an example), so for fixed weights post-hoc bias will also increase as the MAF of variant $i$ increases.)
4. The weighted sum of the covariance between test statistics at marker $i$ and other markers in the gene $j\neq i$ increases. (Note: the covariance between test statistics is related to LD, so for fixed weights post-hoc bias will also increase as LD increases within the gene.)

**A.1.2 Post-hoc variance**

From the form of the conditional distribution of the single-marker statistic $T_{i}$ given the GBT $Q$, it follows that the form of the post-hoc variance (i.e., the variance of $T_{i}$ given $Q$) is:

$$\mathrm{Var}\left( T_{i} \right|Q)=V\left( T_{i} \right)-\frac{\left( w_{i}V\left( T_{i} \right)+\sum_{j\neq i} w_{j}\sigma_{ij} \right)^{2}}{V\left( Q \right)}.$$

Since the second term in this equation $\left( \frac{\left( w_{i}V\left( T_{i} \right)+\sum_{j\neq i} w_{j}\sigma_{ij} \right)^{2}}{V\left( Q \right)} \right)$ will always be positive, we see that conditioning on the GBT $Q$ reduces the variance of the post-hoc single-marker test statistic. The magnitude of this reduction depends on the variance of the GBT, the variance of the single-marker statistic, and the LD structure within the gene.

**A.1.3 Post-hoc MSE**

From the form of the conditional distribution of the single-marker statistic $T_{i}$ given the GBT $Q$, it follows that the form of the post-hoc mean squared error (i.e., the MSE of $T_{i}$ given $Q$) is:

$$\mathrm{MSE}\left( T_{i} \right|Q)=\mathrm{Bia}s^{2}+\mathrm{Var}=\left( \frac{w_{i}V\left( T_{i} \right)+\sum_{j\neq i} w_{j}\sigma_{ij}}{V\left( Q \right)}\mathrm{Bias}\left( Q \right) \right)^{2}+ V\left( T_{i} \right)-\frac{\left( w_{i}V\left( T_{i} \right)+\sum_{j\neq i} w_{j}\sigma_{ij} \right)^{2}}{V\left( Q \right)}= \left( \frac{w_{i}V\left( T_{i} \right)+\sum_{j\neq i} w_{j}\sigma_{ij}}{V\left( Q \right)} \right)^{2}\left( \mathrm{Bias}\left( Q \right)^{2}-V\left( Q \right) \right)+V\left( T_{i} \right).$$

If we did not condition on the GBT $Q$, the MSE of the single-marker statistic would be $\mathrm{MSE}(T_{i})=\mathrm{Bias}\left( T_{i} \right)^{2}+\mathrm{Var}(T_{i})=V(T_{i})$, assuming $T_{i}$ is an unbiased estimator of $\mu_{i}$.

Comparing the two MSEs, we see that conditioning on the GBT $Q$ will increase MSE if $\mathrm{Bias}\left( Q \right)^{2}>V\left( Q \right)$. As mentioned above, calculating single-marker test statistics only after significant Step 1 tests can likely result in large $\mathrm{Bias}(Q)$ due to winner’s curse at the level of the GBT. So, in the post-hoc single-marker estimation setting it is very likely that $\mathrm{Bias}\left( Q \right)^{2}>V\left( Q \right)$ and hence the post-hoc MSE will be larger than if we had not first conducted a GBT.

**A.2 Intuition for behavior of post-hoc statistics in our simulation study**

The previous section provides a general framework for understanding the behavior of post-hoc single marker test statistics. In this section, we consider a specific application to the data-generating model, Step 1 tests, and Step 2 statistics considered in our simulation study.

The bias observed in the difference in minor allele frequencies between cases and controls at marker *i*, $D_{i}= C_{i}^{+}/2N^{+}- C_{i}^{-}/2N^{-}$, after a significant Step 1 gene-based test can be written as $Bias\left( D_{i}|S_{1} \right)= E\left( D_{i}|S_{1} \right)-E\left( D_{i} \right)$, where *S*_1_ indicates that the Step 1 test was significant. In other words, this indicates that the observed bias in the single marker difference in minor allele frequencies, *D_i_*, is the difference between the average value of *D_i_*, conditional on a significant Step 1 test, and the expected value of the unconditional difference in minor allele frequencies.

We begin with the common, reasonable assumption that the number of minor alleles observed at variant $i$ in the cases ($C_{i}^{+}$) and the number of minor alleles observed at variant$i$ in the controls ($C_{i}^{-}$) are distributed according to Binomial distributions:

$$C_{i}^{+}\sim Binomial\left( 2N^{+}, f_{i}^{+} \right)\equiv Binomial\left( 2N^{+}, \lambda_{i}f_{i}^{-} \right)$$

$$C_{i}^{-}\sim Binomial\left( 2N^{-}, f_{i}^{-} \right)$$

Where, $f_{i}^{-}$is the population minor allele frequency for variant *i* in the controls, $f_{i}^{+}$is the minor allele frequency for variant *i* in the cases, $\lambda_{i}$ is the relative risk of the *i*^th^ variant $\left( \lambda_{i}=\frac{f_{i}^{+}}{f_{i}^{-}} \right)$, $N^{+}$ is the total number of cases, and $N^{-}$is the total number of controls. For a rare disease, $f_{i}^{-}\approx f_{i}$, where $f_{i}$ is the overall population minor allele frequency of variant $i$.

Then, letting $\mu_{i}$ and $\sigma_{i}^{2}$ be the mean and standard deviation of the unconditional differences in minor allele frequencies, $D_{i},$we note the following relationships:

$$\mu_{i}=E\left( D_{i} \right)= E\left( \frac{C_{i}^{+}}{2N^{+}}- \frac{C_{i}^{-}}{2N^{-}} \right)= \frac{E\left( C_{i}^{+} \right)}{2N^{+}}-\frac{E\left( C_{i}^{-} \right)}{2N^{-}}=f_{i}^{+}-f_{i}^{-}=\left( \lambda_{i}f_{i}^{-}-f_{i}^{-} \right)=f_{i}^{-}(\lambda_{i}-1)$$

$$\sigma_{i}^{2}=V\left( D_{i} \right)=\frac{V\left( C_{i}^{+} \right)}{\left( 2N^{+} \right)^{2}}+\frac{V\left( C_{i}^{-} \right)}{\left( 2N^{-} \right)^{2}}=\frac{2N^{+}f_{i}^{+}\left( 1-f_{i}^{+} \right)}{\left( 2N^{+} \right)^{2}}+\frac{{2N^{-}f}_{i}^{-}\left( 1-f_{i}^{-} \right)}{\left( 2N^{-} \right)^{2}}=\frac{f_{i}^{+}\left( 1-f_{i}^{+} \right)}{2N^{+}}+\frac{f_{i}^{-}\left( 1-f_{i}^{-} \right)}{2N^{-}}= \left[ \frac{\lambda_{i}f_{i}^{-}\left( 1-\lambda_{i}f_{i}^{-} \right)}{2N^{+}}+ \frac{f_{i}^{-}\left( 1-f_{i}^{-} \right)}{2N^{-}} \right]$$

Where the equality $V\left( D_{i} \right)= \frac{V\left( C_{i}^{+} \right)}{\left( 2N^{+} \right)^{2}}+\frac{V\left( C_{i}^{-} \right)}{\left( 2N^{-} \right)^{2}}$ follows from the assumption that $C_{i}^{+}$ is independent of $C_{i}^{-}$.

If we further assume that we have the same number of cases and controls (so $N=N^{+}= N^{-}$) then

$$\mu_{i}=\left( \lambda_{i}f_{i}^{-}-f_{i}^{-} \right)$$

$$\sigma_{i}^{2}=\frac{1}{2N}[\lambda_{i}f_{i}^{-}\left( 1-\lambda_{i}f_{i}^{-} \right)+ f_{i}^{-}\left( 1-f_{i}^{-} \right)]=\frac{f_{i}^{-}\left[ \lambda_{i}+1 \right]}{2N}-\frac{f_{i}^{- 2}[{\lambda_{i}^{2}}+1]}{2N} \approx\frac{f_{i}^{-}[\lambda_{i}+1]}{2N}$$

The last approximation comes from a first order Taylor expansion of $f_{i}^{-}$around 0 and $\lambda_{i}$ around 1, and demonstrates that variance of $D_{i}$ increases with minor allele frequency and relative risk. This can also be seen in the left panels of Supplemental Figures 3 and 4, which plot $\sigma_{i}^{2}$ versus $f_{i}^{-}$ and $\lambda_{i}$.

In order to gain intuition into the distribution of $Bias\left( D_{i}|S_{1} \right)$, we make the simplifying assumption that minor allele counts $C_{i}^{+}$ and $C_{i}^{-}$ are approximately normally distributed with means $2N^{+}\lambda_{i}f_{i}^{-}$ and $2N^{-}f_{i}^{-}$, respectively, and variances ${2N}^{+}\lambda_{i}f_{i}^{-}\left( 1-\lambda_{i}f_{i}^{-} \right)$ and ${2N}^{-}f_{i}^{-}\left( 1-f_{i}^{-} \right)$, respectively. Then $D_{i} \sim N(\mu_{i}, \sigma_{i}^{2})$. Further, we assume that the *m* variants in the gene have independent genotypes.

While approximate normality is reasonable for large sample sizes and minor allele frequencies that are not too close to 0, it is not strictly true for small sample sizes and rare variants. Additionally, independence fails to account for linkage disequilibrium. However, the patterns observed assuming approximate normality and independence of variants in a gene lead to insights confirmed in simulated data for small sample sizes (see *Results* for details).

Recall that the test statistic for a Step 1 burden test is $Q_{bw}\approx\sum_{i=1}^{m} {w_{i}D}_{i}$. Given that $D_{i}\dot{\sim}N\left( \mu_{i},\sigma_{i}^{2} \right)$, and $D_{i}\perp D_{j} \forall i \neq j,$ then $Q_{b}\dot{\sim}N(\sum_{i} w_{i}\mu_{i}, \sum_{i} w_{i}^{2}\sigma_{i}^{2})$. We note that if $X \sim N(\mu_{x}, \sigma_{x}^{2})$ and Y$\sim N(\mu_{y}, \sigma_{y}^{2})$ , then $X|Y=y \sim(\mu_{x}+\frac{\sigma_{yx}^{2}}{\sigma_{y}^{2}}\left( y-\mu_{y} \right), \sigma_{xx}^{2}-\frac{\sigma_{yx}^{4}}{\sigma_{\mathrm{yy}}^{2}})$. Thus, we have $E\left( D_{i} \right|Q_{bw}=q)= \mu_{i}+\frac{Cov\left( D_{i},Q_{bw} \right)}{Var\left( Q_{bw} \right)}\left( q-\sum w_{i}\mu_{i} \right)=\mu_{i}+\frac{{w_{i}\sigma}_{i}^{2}}{\sum w_{i}^{2}\sigma_{i}^{2}}\left( q-\sum w_{i}\mu_{i} \right)$. Combining yields the following result:

$$Bias\left( D_{i}|Q_{bw}=q \right)= E\left( D_{i}|Q_{bw}=q \right)-E\left( D_{i} \right)= \frac{w_{i}\sigma_{i}^{2}}{\sum{w_{i}^{2}\sigma}_{i}^{2}}\left( q-\sum w_{i}\mu_{i} \right)=\left( \frac{V\left( variant \right)}{V\left( gene \right)} \right)\times Weight\left( variant \right)\times Bias\left( gene \right)$$

This demonstrates that the bias in difference in minor allele frequencies for a single variant is proportional to the variance of the difference in minor allele frequencies for that variant, which we showed above increases approximately linearly with allele frequency. This explains why we see more bias in common variants than rare variants (see *Results* for details).

We use a similar argument to find the bias in $D_{i}^{2}= \left( f_{i}^{+}- f_{i}^{-} \right)^{2}$. First, we note that

$$E\left( D_{i}^{2} \right)=E^{2}\left( D_{i} \right)+V\left( D_{i} \right)= \mu_{i}^{2}+\sigma_{i}^{2}$$

$$V\left( D_{i}^{2} \right)=E\left( D_{i}^{4} \right)-E^{2}\left( D_{i}^{2} \right)$$

If we assume that $D_{i}\dot{\sim}N\left( \mu_{i},\sigma_{i}^{2} \right)$, then

$$V\left( D_{i}^{2} \right)=3\sigma_{i}^{4}+6\sigma_{i}^{2}\mu_{i}^{2}+\mu_{i}^{4}-\mu_{i}^{4}-\sigma_{i}^{4}-2\sigma_{i}^{2}\mu_{i}^{2}= {2\sigma}_{i}^{2}(\sigma_{i}^{2}+2\mu_{i}^{2})$$

Further, if we assume that $D_{i}^{2}$ and $Q_{sw}=\sum_{i=1}^{m} w_{i}D_{i}^{2}$ are approximately normally distributed and that the *m* variants in the gene have independent genotypes (so $Cov\left( D_{i}^{2}, D_{j}^{2} \right)=0 \forall i\neq j$), then we can apply the identity above and we get

$$Bias\left( D_{i}^{2}|Q_{sw}=q \right)= E\left( D_{i}^{2}|Q_{sw}=q \right)-E\left( D_{i}^{2} \right)=\left( \frac{V\left( variant \right)}{V\left( gene \right)} \right)\times Weight\left( variant \right)\times Bias\left( gene \right)$$

Where, $V\left( variant \right)$=$2\sigma_{i}^{2}(\sigma_{i}^{2}+2\mu_{i}^{2})$, $V\left( gene \right)=\sum w_{i}^{2}{2\sigma}_{i}^{2}(\sigma_{i}^{2}+2\mu_{i}^{2})$, $Weight\left( variant \right)=w_{i}$, and $Bias\left( gene \right)=q-\sum w_{i}(\mu_{i}^{2}+\sigma_{i}^{2})$.
